# Fasting-mimicking diet causes hepatic and blood markers changes indicating reduced biological age and disease risk

**DOI:** 10.1038/s41467-024-45260-9

**Published:** 2024-02-20

**Authors:** Sebastian Brandhorst, Morgan E. Levine, Min Wei, Mahshid Shelehchi, Todd E. Morgan, Krishna S. Nayak, Tanya Dorff, Kurt Hong, Eileen M. Crimmins, Pinchas Cohen, Valter D. Longo

**Affiliations:** 1 Longevity Institute, Leonard Davis School of Gerontology, University of Southern California, Los Angeles, CA 90089 USA; 2 Department of Pathology, Yale School of Medicine, New Haven, CT 06519 USA; 3 Ming Hsieh Department of Electrical Engineering, Viterbi School of Engineering, University of Southern California, Los Angeles, CA 90089 USA; 4 Norris Comprehensive Cancer Center, Keck School of Medicine, University of Southern California, Los Angeles, CA 90033 USA; 5 Center of Clinical Nutrition and Applied Health Research, Keck School of Medicine of USC, Los Angeles, CA 90033 USA; 6 Center on Biodemography and Population Health, University of California Los Angeles and University of Southern California, Los Angeles, CA 90089 USA; 7 AIRC Institute of Molecular Oncology, Italian Foundation for Cancer Research Institute of Molecular Oncology, 20139 Milan, Italy

**Keywords:** Biomarkers, Risk factors

## Abstract

In mice, periodic cycles of a fasting mimicking diet (FMD) protect normal cells while killing damaged cells including cancer and autoimmune cells, reduce inflammation, promote multi-system regeneration, and extend longevity. Here, we performed secondary and exploratory analysis of blood samples from a randomized clinical trial (NCT02158897) and show that 3 FMD cycles in adult study participants are associated with reduced insulin resistance and other pre-diabetes markers, lower hepatic fat (as determined by magnetic resonance imaging) and increased lymphoid to myeloid ratio: an indicator of immune system age. Based on a validated measure of biological age predictive of morbidity and mortality, 3 FMD cycles were associated with a decrease of 2.5 years in median biological age, independent of weight loss. Nearly identical findings resulted from  a second clinical study (NCT04150159). Together these results provide initial support for beneficial effects of the FMD on multiple cardiometabolic risk factors and biomarkers of biological age.

## Abstract (web summary)

Fasting mimicking diets (FMDs) can promote healthy aging in animal models but effects on human healthspan remain elusive. Here the authors provide support for beneficial effects of the FMD on cardiometabolic risk factors and biological age biomarkers.

## Introduction

Metabolic syndrome, classified by co-occurrence of three of the following symptoms: insulin resistance, dyslipidemia, elevated inflammation and CRP levels, and abdominal obesity and affecting approximately 37% of the US population and 49% of those over 60, increases the risk of cardiovascular morbidity and mortality^1^. Excess calorie consumption and the resulting accumulation of fat in the liver cause the development of non-alcoholic fatty liver disease (NAFLD) in up to 75% of people with obesity^2^, which can lead to non-alcoholic steatohepatitis (NASH)^3^, and is projected to become the leading cause of liver related morbidity and mortality within the next 20 years. Research over the past decade has demonstrated that obesity and the associated changes in insulin, lipids and inflammation accelerates the rate of aging in the liver, brain, adipose tissue and other organs^4–8^, possibly by acting on molecular hallmarks of aging^9^. In addition to metabolic dysregulation, another feature of aging in both rodents and humans is the altered composition and function of the immune system, also known as immunosenescence^10^, which with time, leads to a decline in immune function, increased vulnerability to infectious diseases, diminished responses to vaccination, and an increased susceptibility to age-related inflammatory diseases^11^.

Because age-dependent dysfunction in different types of cells is at the center of NAFLD, diabetes, autoimmunity, cancer, neurodegeneration, cardiovascular disease etc., intervening on the aging process has the potential to ameliorate or prevent many human diseases, as demonstrated in animal models, by slowing down cellular deterioration but also by replacing old and damaged cells and intra-cellular components with newly generated or functional ones^12^. Of note, intervening on the aging process is not an approach that should be restricted to the old but could instead start in younger adults since aging and age-related chronic diseases results from cumulative cellular and molecular damage or changes^13^. For example, changes in gut microbiota, as a result of dietary habits and other extrinsic factors, can promote inflammation and thereby possibly modulate the healthspan of elderly people^14^. Everyday nutrition and particularly protein as well as carbohydrate levels and sources are known to play a pivotal role in accelerating the aging process and mortality in rodents and possibly humans^15–17^. Notably, not only the level of calories consumed and the macronutrient composition influence health and lifespan, but the number of hours for which meals are consumed. Providing a limited window of time in which rodents consume an otherwise unlimited amount of food, known as time-restricted eating (TRE), is associated with improvements in health and longevity^15,18^. Similarly, intermittent fasting (IF, an eating pattern that cycles between periods of eating and fasting per week) including alternate day fasting (ADF, days of low-calorie consumption or complete fasting alternated with days of either ad libitum food consumption or feasting) promotes protection against multiple diseases and induces lifespan extension^19,20^. Periodic water-only fasting (PF, total food abstinence for several days without limiting hydration) has several benefits in preclinical studies such as weight loss, improved insulin sensitivity, autophagy activation, cell renewal, and anti-cancer effects, but is associated with some safety and compliance concerns which may be responsible for the limited contribution of PF to standard medical practice^15,21–29^. The periodic use of a fasting-mimicking diet (FMD; a plant-based, low-calorie and low-protein 5-day lasting dietary intervention) followed by a normal diet has positive effects on both cellular function and healthspan. Unlike for chronic interventions that do not involve a sufficient length of fasting to cause organ and cellular atrophy, cycles of long-term fasting/FMD and -refeeding allow mouse cells and organs to activate breakdown processes that target dysfunctional cells^30,31^ followed by either regenerative or reprogramming phases during the re-feeding period in which new or more functional cells are generated^30,32^. In C57Bl6 mice, when started at middle-age, FMD cycles result in lifespan extension as well as a 45% reduction in tumor incidence and a major reduction in inflammatory diseases^33^. In a previously published clinical trial we compared study participants who followed 3 months of a standard diet to study participants who consumed the FMD for 5 consecutive days per month for 3 months, but otherwise also consumed a standard diet^34^. Three FMD cycles reduced body weight, trunk and total body fat, blood pressure, and decreased insulin-like growth factor 1 (IGF-1) without causing serious adverse effects. In a post-hoc analysis, body mass index, blood pressure, fasting glucose, IGF-1, triglycerides, total and low-density lipoprotein cholesterol, and C-reactive protein were more strongly reduced in participants with high baseline levels of these risk factors^34^.

Here, we test the hypothesis that FMD cycles improve the levels of multiple markers of aging thus reducing biological age as measured by a set of validated blood markers and by other cellular and metabolic measurements. We report on the secondary outcome measures of the FMD-trial which are biomarkers associated with aging or age-related diseases, and metabolic syndrome, including visceral and hepatic fat, lymphoid/myeloid ratios, and blood markers, which were not investigated in the original report. These measurements allow for an exploratory analysis to estimate biological age in participants in two different clinical trials (NCT02158897, NCT04150159) before and after they completed 3FMD cycles.

## Results

### Study participants’ baseline data

**Fig. 1:**
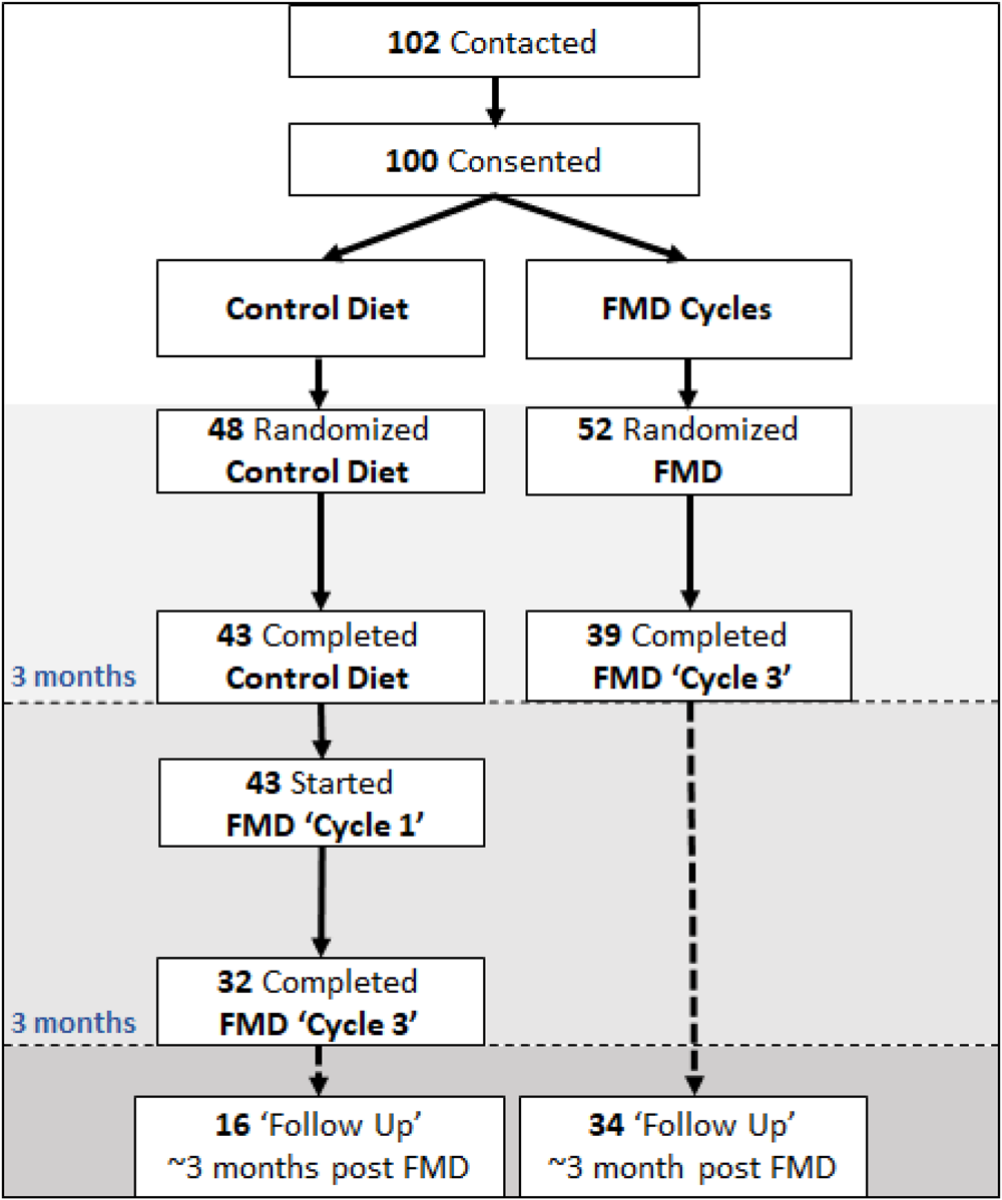
CONSORT diagram. Consolidated Standards of Reporting Trials (CONSORT) diagram of 102 contacted study participants of which 100 were enrolled into the study two arms. Arm 1 (*n* = 48), the control group, maintained their normal caloric intake for a 3-month monitoring period. Data were collected at enrollment and again after 3 months. Participants in arm 2 (*n* = 52) started the FMD after randomization. The FMD was provided for 5 days per month for three consecutive cycles. Data were collected at enrollment, at the completion of the first FMD cycle but before resuming normal dietary intake, and also on average 5 days after study participants resumed their normal diet after the final FMD cycle. After the initial 3-month period, study participants in arm 1 also started the FMD. An optional follow-up visit in the clinic for analysis was offered to all participants about 3 months after the completion of the third FMD cycle. From Wei, Min et al. “Fasting-mimicking diet and markers/risk factors for aging, diabetes, cancer, and cardiovascular disease.” Science translational medicine vol. 9377 (2017): eaai8700. doi:10.1126/scitranslmed.aai8700. Reprinted with permission from AAAS.

Primary outcome measures for this trial (clinicaltrials.gov/NCT02158897) were to establish the effect of the FMD on metabolic syndrome and biomarkers associated with aging or age-related diseases. For this randomized trial, the sample size of 100 total study participants was based on the detection of a 25% reduction in mean insulin-like growth factor-1 (IGF-1), with a two-sided α of 0.05 and 70% power. The estimated control group mean (SD) IGF-1 of 194 (97) used published data on males and females aged 26 to 40 years^35^. The baseline characteristics for all study participants were previously reported^34^. In brief, 100 study participants were randomized and assigned to either arm 1 (*n* = 48) or arm 2 (*n* = 52). At enrollment, study participants in the two arms were comparable for age, sex, race, and body weight (Table 1). The participants in control arm 1 were asked to continue their normal diet for 3 months, whereas participants in intervention arm 2 started the FMD cycles (Fig. 1). After 3 months, 43 study participants from the control arm were crossed over to the FMD intervention. We compared the changes in trial outcomes between the two groups who completed three FMD cycles (*n* = 39 FMD randomized arm 2 and *n* = 32 after arm 1 crossover to FMD) using sensitivity analysis (Table S1). Three FMD cycles had comparable effects in arm 1 (after crossover) and arm 2 with the exception of high-density lipoprotein (HDL) cholesterol, for which a greater reduction was observed in arm 2 (*P* = 0.03), and a decrease in absolute lean body mass, which was observed in arm 2 but not in arm 1^34^. An optional follow-up visit about 3 months after completion of the final FMD cycle was offered. Results from this study were published previously^34^. Here we now report secondary as well as exploratory outcomes from this trial. 15 participants (10 in arm 2 and 5 in arm 1 after crossing over to the FMD intervention), 10 of which were affected by overweight, volunteered to have their body fat composition measured by magnetic resonance imaging (MRI) prior to the FMD intervention and after the 3rd FMD cycle. The 15 volunteers, who were not pre-selected based on any demographic/biometric and biomarker parameters, were not significantly different from the rest of the study population with regards to age (non-MRI 43.0 vs. MRI 41.3 years; *p*-value 0.62; 95% CI −8.496 to 5.110), height (non-MRI 166.9 vs. MRI 166.9 cm; *p*-value 0.98; 95% CI −5.328 to 5.467), or body mass index (BMI, kg/m^2^; non-MRI 26.9 vs. MRI 28.1 BMI; *p*-value 0.35; 95% CI −1.420 to 4.030), but the MRI volunteer cohort had a more even gender distribution (53% male and 47% female) compared to the non-MRI volunteers (34% male and 66% female).

**Table 1:** Characteristics of all study participants at enrollment^a^

Characteristic	Arm 1 (*N* = 48)	Arm 2 (*N* = 52)
Sex – no. of subjects (%) Male Female	18 (37.5) 30 (62.5)	19 (36.5) 33 (63.5)
Race or ethnic group – no. of subjects (%)^b^ White Black Hispanic Asian Other	26 (54.2) 2 (4.2) 13 (27.1) 6 (12.5) 1 (2.1)	25 (48.1) 5 (9.6) 14 (26.9) 7 (13.5) 1 (1.9)
Age (years)	42.2 ± 12.5	43.3 ± 11.7
Weight (kg)	77.0 ± 15.9	74.3 ± 16.6
Education (years)	16.7 ± 2.8	16.6 ± 2.3
Smoking Status – no. of subjects (%) Never smoked Former smoker Current smoker	29 (60.4) 13 (27.1) 6 (12.5)	39 (75.0) 9 (17.3) 4 (7.7)
Body-mass Index^c^ Mean <25 - no. of subjects (%) 25 to 30 - no. of subjects (%) > 30 - no. of subjects (%)	27.8 ± 5.1 17 (35.4) 18 (37.5) 13 (27.1)	26.6 ± 4.9 20 (38.4) 21 (40.4) 11 (21.2)
Systolic Blood Pressure (mmHg)	117.2 ± 12.3	117.2 ± 13.0
Diastolic Blood Pressure (mmHg)	75.6 ± 9.2	75.2 ± 7.8
Triglycerides (mg/dL)	104.0 ± 64.6	84.7 ± 37.2
Cholesterol (mm/dL) Total Low-density lipoprotein High-density lipoprotein	197.5 ± 39.6 114.5 ± 36.1 62.2 ± 16.4	185.7 ± 36.6 110.3 ± 61.6 65.2 ± 18.1

^a^Plus-minus values are mean ± SD rounded to the nearest tenth.

^b^The race or ethnic group was assigned by the subjects themselves.

^c^The body-mass index is the weight in kilograms divided by the square of the height in meters.

Additionally, blood samples from FMD study participants in a second trial (clinicaltrials.gov/NCT04150159), focused on the use of an FMD (same as for the trial above) to reduce cardiometabolic risk factors, were included to measure hypothetical effects of the FMD on biological age, disease risk, and life-expectancy^36^. 44 eligible (defined as BMI ≥ 28, and diagnosed with either endothelial dysfunction (reactive hyperemia index ≤ 2.0), or low small resistance artery compliance (AC2 ≤ 5.0)) study participants (73% female, 54.9 ± 12.2 years-old, BMI 33.9 ± 4.8), were instructed to consume the FMD for 4 consecutive months. Primary (endothelial function and arterial compliance) and secondary outcome measures (blood pressure, serum lipids, glucose, cardiovascular biomarkers, body weight, BMI, and body composition) of this trial are presented elsewhere^36^. We are here using baseline as well as values after the 3^rd^ FMD cycle to match the time points of trial NCT02158897.

### FMD decreases abdominal and hepatic fat

**Fig. 2:**
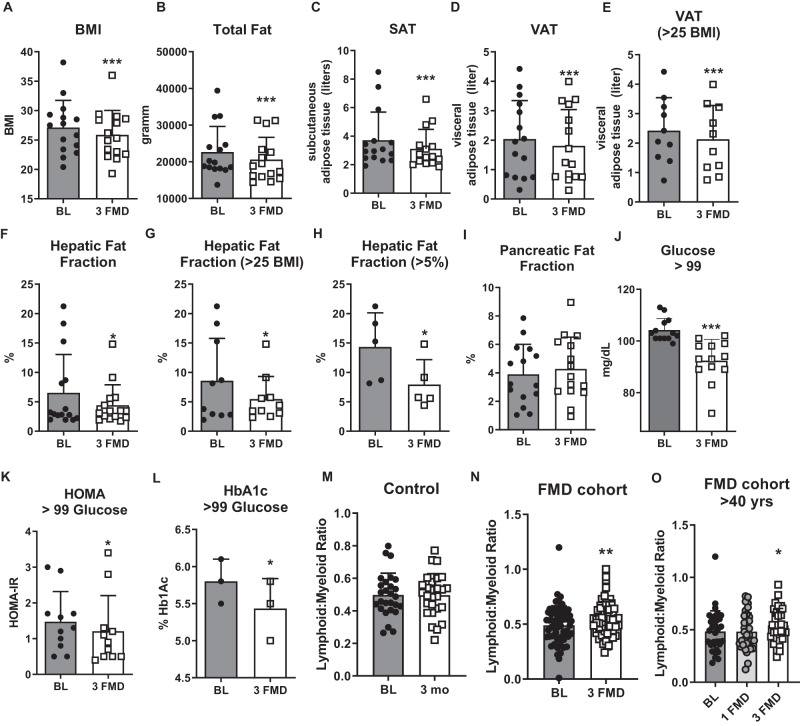
Fasting-mimicking decreases the hepatic fat fraction and diabetes risk markers, and increases the lymphoid to myeloid ratio. **A** Body mass index BMI; *N* = 15 biologically independent samples. *p* = 0.0002; Baseline (BL)= gray bar, 3 fasting-mimicking diet (FMD) cycles= white bar. **B** Total fat in grams; *N* = 15 biologically independent samples measured by DEXA. *p* = 0.0021. **C** Subcutaneous adipose tissue (SAT) in liters; *N* = 15 biologically independent samples. *p* = 0.0084. **D** Visceral adipose tissue (VAT) in liters; *N* = 15 biologically independent samples. *p* = 0.0017. **E** Visceral adipose tissue (VAT) in liters for study participants affected by overweight and obesity; *N* = 10. *p* = 0.0030. **F** Hepatic fat fraction (HFF) in percent for all study participants (*N* = 15, *p* = 0.0495) and **G** HFF in study participants affected by overweight and obesity (*N* = 10, *p* = 0.0465) and (**H**) for study participants with fatty liver described as >5% HFF (*N* = 5, *p* = 0.0197). **I** Pancreatic fat fraction (PFF) for *N* = 15 study participants. *p* = 0.2778. **J** Serum glucose in pre-diabetic study participants at baseline and after 3 FMD cycles; *N* = 13. *p* = < 0.0001. **K** Homeostatic model assessment to measure insulin resistance (HOMA-IR) in pre-diabetic study participants; *N* = 11. *p* = 0.0455. **L** Hemoglobin A1c (HbA1c) levels in pre-diabetic study participants; *N* = 3. *p* = 0.0315. **M** The lymphoid to myeloid ratio in study participants that maintained a normal diet; *N* = 27. *p* = 0.9723 Baseline (BL)= gray bar, 3 month follow-up (3mo)= white bar. **N** Following 3 monthly cycles of a fasting-mimicking diet, the lymphoid to myeloid ratio was increased for all participants that completed the trial; *N* = 61. *p* = 0.0049. **O** The lymphoid to myeloid ratio at baseline for study participants older than 40 years at baseline (BL > 40; *N* = 34; dark gray bar), after completion of the 1st FMD cycle (*p* = 0.9585; 1FMD light gray bar) and 3–5 days of resuming normal food intake after the 3rd FMD cycle (*p* = 0.0356, 3 FMD white bar). Mean ± SD. Paired two-tailed *t*-test. *p < 0.05, **p < 0.01, ***p < 0.001. **C**–**I** measured by MRI. Source data are provided as a Source Data file.

Abdominal obesity increases the risk of developing non-alcoholic fatty liver disease as well as cardiovascular morbidity and mortality. We previously reported that participants in the FMD study displayed reduced weight and BMI, in part due to a reduction in total body fat and truncal fat measured by dual-energy x-ray absorptiometry (DEXA)^34^. In mice, the FMD reduces visceral adipose tissue but not subcutaneous adipose tissue based on magnetic resonance imaging (MRI)^33^. Here, we measured the abdominal fat distribution in 15 volunteers using MRI. Three cycles of the FMD reduced their BMI (Fig. 2A, kg/m^2^; *p* = 0.0002; 95% CI 0.734 to 1.8) and total body fat (Fig. 2B, *p* = 0.002; 95% CI 875 to 3199). For all 15 study participants and in those with a BMI > 25, subcutaneous (SAT; *p* = 0.008; 95% CI 0.179 to 1.016 and) and visceral adipose tissue (VAT; p = 0.002; 95% CI 0.103 to 0.358; VAT in overweight N = 10, p = 0.003; 95% CI 0.129 to 0.459) was reduced after the 3rd FMD cycle compared to their baseline evaluation (Fig. 2C–E). The hepatic fat fraction (HFF) was also reduced (Fig. 2F, *p* = 0.049; 95% CI 0.005 to 4.195). Because previous studies indicate that the normal hepatic fat content in adults is less than 5%^37^, we used a cut-off value of 5% for diagnosing hepatic steatosis in our cohort. In 10 study participants affected by overweight, with only 5 qualifying as having hepatic steatosis (% HFF of over 5%, 50% incidence) HFF was reduced from 8.57 ± 7.2% to 5.45 ± 3.86% (Fig. 2G, *p* = 0.047; 95% CI 0.060 to 6.178). Notably, in those 5 study participants with HFF > 5%, the percentage of liver fat was reduced from 14.32 ± 5.8% to 7.94 ± 4.22%, a nearly 50% reduction (Fig. 2H, *p* = 0.02; 95% CI 1.676 to 11.09). In contrast, the percentage of pancreatic fat fraction remained unchanged (Fig. 2I, *p* = 0.278; 95% CI −1.104 to 0.342). These results, although based on a small subset of trial volunteers, are in line with previous data indicating that the FMD can decrease abdominal obesity and provide preliminary data on the potential for the FMD as a treatment for hepatic steatosis.

### FMD and diabetes risk

Approximately 85–90% of people with type 2 diabetes are affected by either overweight or obesity^38^. We previously showed that three FMD cycles can restore glucose levels in pre-diabetic study participants with fasting glucose >99 mg/dl (Fig. 2J, *N* = 13) to a normal range, which was preserved for at least 3 months following the intervention^34^. Here, we used the homeostatic model assessment to measure insulin resistance (HOMA-IR)^39^ prior to and after completion of the third FMD cycle in pre-diabetic study participants. HOMA-IR was reduced from 1.473 ± 0.85 to 1.209 ± 0.99 (*p* = 0.046, *N* = 11; Fig. 2K; −95% CI 0.521 to −0.006), and Hemoglobin A1c levels were reduced from 5.8 ± 0.3 to 5.43 ± 0.404 (p = 0.032, N = 3; Fig. 2L; 95% CI −0.654 to −0.079). Although the number of examined study participants is small, taken together with our previous study these results indicate that the FMD can reduce insulin resistance and help restore normal glucose tolerance in individuals that had fasting glucose levels indicative of pre-diabetes.

### Effects of FMD on the immune profile

Aging causes an altered production and function of immune cells, a phenomenon known as “immunosenescence,” manifested in part as a shift in the lymphoid-to-myeloid ratio and elevated incidence of anemia and myeloid malignancies^33,40,41^. In humans, the decline in lymphopoiesis with age is associated with the transcriptional up-regulation of genes that specify myeloid-lineage differentiation in hematopoietic stem cells; this myeloid-skewing of aged HSCs might contribute to the incidence increase of myeloid disorders in the elderly^42^. In mice, FMD cycles started at middle age cause a rejuvenation of the blood profile including a partial reversal of age dependent decline in the lymphoid-to-myeloid ratio (L/M)^33^. Here, we measured the L/M in study participants that maintained their regular diet (arm 1 prior to cross over) and in all study participants that completed 3 cycles of the FMD. No change in the L/M ratio was detectable in study participants on the control diet (Fig. 2M, *p* = 0.972; 95% CI −0.060 to 0.059). However, a significant improvement (*p* = 0.005) in the L/M ratio was measured in all study participants receiving 3 FMD cycles (Fig. 2N; 95% CI 0.019 to 0.101). Similarly, the L/M ratio increased in over 40-year-old study participants following the 3 FMD cycles and after returning to the normal diet (Fig. 2O, *p* = 0.036; 95% CI 0.004 to 0.113). Thus, 3 FMD cycles in humans, promote changes consistent with previous results in mice indicating rejuvenating effects on the immune system.

### FMD and biological age

In rodents, FMD cycles reduce cancer incidence, improve healthspan and increase median lifespan^33^. In humans, surrogate markers can function as proxies to evaluate the impact of interventions on health or lifespan^43^. Biological age estimates have been developed to approximate the level of aging and subsequent morbidity and mortality risk for an individual. Estimates based on clinical chemistry typically combine multi-system biomarkers (here: albumin, alkaline phosphatase, serum creatinine, C-reactive protein, Hba1c, systolic blood pressure, and total cholesterol) into a single variable meant to capture the level and rate of organismal aging^44–48^ (Table S2). The FMD-induced changes for the individual markers were reported previously^34^.

**Fig. 3:**
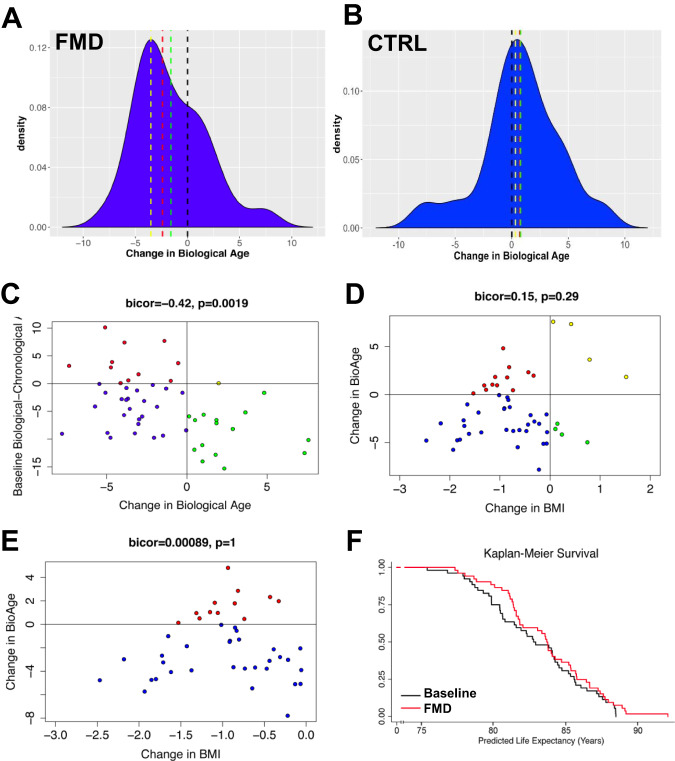
FMD reduces biological age between enrollment and completion of trial independent of weight loss. **A** On average biological age decreased by approximately 1.5 years (green line) between baseline and after three FMD cycles; however, the median decrease was nearly 2.5 years (red line), while the modal decrease was just over 3.5 years (yellow line) in the 52 study participants. **B** Biological age at baseline and three month follow-up did not change for *n* = 16 control study participants. **C** The level of change in biological age was inversely related to the baseline difference in biological relative to chronological age, suggesting that those who had the highest aging rates at baseline showed the greatest improvements. Points are color coded such that red denotes study participants with accelerated biological ages who showed improvements following three cycles of FMD (*N* = 11), blue denotes study participants with decelerated biological ages who showed improvements following three cycles of FMD (*N* = 25), yellow denotes study participants with accelerated biological ages who showed worsening biological age following three cycles of FMD (*N* = 1), and green denotes study participants with decelerated biological ages who showed worsening biological age following three cycles of FMD (*N* = 15). **D** Change in biological age between baseline and completion of three cycles of FMD is not significantly associated with declines in BMI during this time (*r* = −0.075, *p* = 0.35). **E** This is even more apparent when limiting the sample to those who did experience reductions in BMI following 3 cycles of FMD. **C**–**E** Median-based biweight midcorrelation was used to test for association. Significance was assessed with unadjusted two-sided *p*-values. **F** Predicted median life expectancy based on chronological and biological ages was increased following three cycles of FMD. The Kaplan–Meier plot depicts predicted survival assuming an age of death equivalent to predicted median life expectancy based on each study participants biological and chronological age at Baseline and following 3 cycles of FMD. Source data are provided as a Source Data file.

Here we used a biological age method, previously shown to be a better predictor of remaining life expectancy than chronological age, using a large nationally representative human sample (NHANES)^45^. This measure has further been validated in regards to its correlation with functioning parameters and facial age among a birth cohort of 38 year-olds^46,48^ and was used as a clinical outcome in the “Comprehensive Assessment of Long-Term Effects of Reducing Intake of Energy” (CALERIE) trial. Using this measure, we found that at enrollment the majority of participants had biological ages that were estimated to be younger than their chronological ages, suggesting that on average they may be healthier than the average American. After 3 FMD cycles, median biological age in the 52 study participants decreased by nearly 2.5 years (Fig. 3A, *p* = 0.0007, Wilcoxon Rank Sum Scores (Table S3)). This suggests that study participants who completed 3 cycles of the FMD had profiles which appear biologically younger than they did at baseline.

Biological age at baseline and at the three-month follow-up was also calculated for 19 control study participants, as well as 34 participants treated with FMDs in a second independent trial (NCT04150159) by applying the same equation. At baseline, the 19 study participants had a mean chronological age of 47.6 years and a mean biological age of 45.3 years, suggesting that, like the FMD cohort, they were also somewhat healthier than the average U.S. population. Over the intervention period control study participants exhibited a non-significant average biological age increase of 0.78 years (SD = 3.57; *p* = 0.76; Fig. 3B). In agreement with the results in trial NCT02158897, NCT04150159 produced equivalent results, such that the median decrease in biological age after 3 FMD cycles was 2.7 years (Fig. S3A). Pooling the samples from both FMD trials (*N* = 86) results in a median decrease in biological age of 2.6 years (Fig. S3B).

Notably not all study participants who underwent FMD cycles displayed reduced biological age as 16 out of 52 (30.7%) study participants in trial NCT02158897, and 8 out of 34 (21%) study participants in trial NCT04150159 who underwent FMD cycles exhibited increased biological ages. However, these percentages were much smaller than those for the control group in which 12 out of 19 (63.1%) of study participants exhibited increased biological ages. Figure S1A shows the association between baseline characteristics and the probability of being a non-responder (increased biological age) using data from the NCT02158897trial: baseline systolic blood pressure, CRP, and creatinine were inversely associated with the odds of being a non-responder, such that those who were healthier for these parameters at baseline were more likely to increase biological age following FMD. No significant differences were found as a function of baseline age, sex, BMI, Hba1c, cholesterol, alkaline phosphatase, or albumin. Upon further exploration (Fig. S1B), we found that systolic blood pressure (*p* = 0.006) and CRP levels (*p* = 0.0001) were clearly distinct between the responders and non-responders at baseline. This may suggest that the apparent increased biological age was due to a re-normalization or acute changes of CRP or blood pressure for those who exhibited very low baseline levels. Because both blood pressure and CRP can increase acutely and for reasons unrelated to chronic conditions (white coat hypertension, infections, etc.), it is possible that relatively small increases may have caused an apparent but temporary increase in biological age in those healthier 16 study participants. Another possible explanation is that results may reflect regression towards the mean in which outliers with very low blood pressure and/or CRP at baseline—perhaps due to a technical or biological anomaly—may move closer to the average during follow-up.

The individual changes in biological-chronological age between baseline and after 3 FMD cycles are shown in Supplementary Fig. S2. The change in biological-chronological age as a function of baseline biological-chronological age demonstrates a trend of regression towards the mean, such that study participants with the highest baseline biological ages exhibited the biggest changes after the FMD (Fig. 3C).

### Weight loss alone does not account for the decrease in biological aging

Obesity remains a risk-factor for the early onset of many age-related diseases. Weight loss can reduce blood pressure, insulin resistance, as well as the risk for cancer and cardiometabolic diseases^49–51^. When assessing whether changes in weight were associated with the change in biological age, we found no association (bicor = 0.15, *p* = 0.29, Fig. 3D). Similarly, the correlation between change in BMI and change in biological age among participants who had not gained weight following FMD was also not significant (Fig. 3E), suggesting that greater weight loss was not driving greater decreases in biological age. To further determine whether the changes in markers and biological age in the on average normal weight study participants studied in the clinical trial are a result of weight loss caused by the FMD cycles, we reanalyzed our data, adjusting for changes in weight. When comparing these weight adjusted biological ages (Table S3), we still find a decrease post-intervention that cannot be explained by weight loss alone (*p* = 0.029). This was again validated in trial NCT04150159, which showed even stronger results after considering the effect of weight loss on the change in biological age (*p* < 0.001, Table S3). In summary, these simulations suggest that although weight loss may contribute to changes in variables used to estimate biological age markers, weight loss alone does not explain the effects of FMD cycles on biological age and disease risk.

Next, we used the original sample to assess the association between the change in biological age and in the individual biomarkers that make up the biological age estimate to determine whether changes in a single marker was driving the changes (Supplementary Fig. S3). Overall, there was a significant association of changes in biological age with changes in four of the seven markers—albumin (bicor = 0.37, *p* = 0.007), CRP (bicor = 0.63, *p* < 0.0001), systolic blood pressure (bicor = 0.64, *p* < 0.0001), and Hba1c (bicor = 0.35, *p* = 0.011) after completion of the third FMD cycle; suggesting no single marker alone was responsible for the declines in biological age, but rather this was the result of  a system-level improvement.

We then restricted the sample to at-risk study participants (defined as having at least one of the following: BMI > 25, elevated CRP, fasting glucose >99, and systolic hypertension) and re-evaluated the changes in biological age. We found that these individuals experienced an even greater decrease in biological age—from 43.3 to 40.4 years in biological age—after completion of 3 FMD cycles (*p* = 2.8e-5; Table S3). We then examined the association between BMI changes and the individual biomarkers in the at-risk study participants (Supplementary Fig. S4). Overall, there was no significant association of changes in BMI and the observed changes in each individual biological age marker, aside from a positive association for cholesterol in study participants with elevated CRP levels at baseline (bicor = 0.55, *p* = 0.001; Supplementary Fig. S4B) after completion of the third FMD cycle.

### FMD reduces biological age and increases predicted life expectancy

A reduction in biological age should be associated with a reduction in morbidity risk, which in turn should result in increased healthspan and lifespan. We therefore used the NHANES III dataset to fit mortality models from which we could estimate the predicted life expectancy based on biological age at baseline and following completion of 3 FMD cycles. At baseline, participants in the first trial had an average predicted median life expectancy of 82.93 years ± 3.47, which increased to 83.73 ± 3.32 after 3 FMD cycles. Given that life expectancy estimates represent direct transformations of the biological age estimates, the same level of significance shown for changes in biological age applies (*p* = 0.0007). Kaplan–Meier survival curves assuming median predicted life expectancy as the time of death were plotted for all participants that completed the FMD cycles. Whereas life expectancies in the highest ranges (i.e., oldest chronological age at death) did not show much difference between pre- and post-intervention (Fig. 3F), the FMD cycles caused changes associated with a delay in early deaths, reflected by a divergence in the Kaplan–Meier curves prior to age 82. This is consistent with a similar observation we made in middle aged mice maintained on bi-weekly FMD cycles throughout their adult life. Similar results were found for participants in the second trial, such that at baseline, median life expectancy increased from 81.5 years ± 3.06 to 83.1 ± 3.41 following 3 FMD cycles in the hypertension trial (Fig. S5C). The pooled results for all 86 study participants that completed 3 FMD cycles indicate an increase in median life expectancy from 82.2 years ± 3.52 to 83.5 ± 3.41 (Fig. S5D).

Because the Kaplan–Meier survival curve was based on a single sequence of 3 consecutive cycles of the FMD and does not address the fact that biological age markers would eventually return to the baseline levels as suggested by our previous clinical trial, a simulation was conducted to estimate the potential effects of continued 3 cycles per year of the FMD undertaken annually until age 70. Baseline Biological age and chronological age for NHANES IV participants ages 20–59 (*n* = 7733) was used as the starting point—such that results would represent the effect that the FMD may have on the general population of participants when started at age 20, 30, 40, and 50, respectively, in the NHANES IV sample. A model calculated from the clinical trial data was used to forecast the effect of each annual 3 FMD cycles as a function of biological age and chronological age at the start of each cycle (Fig. S6A). As mentioned previously, FMD cycles had the biggest impact on those with higher than expected biological ages (i.e., accelerated agers). Therefore, in the simulation, as individuals’ biological ages were estimated to improve year-after-year, the decrease in biological age associated with each 3 cycles of FMD became less dramatic—suggestive of a floor effect. For instance, the decline in biological age is fairly rapid at the onset of the simulation (especially at older ages). However, it eventually reaches a point of stabilization such that individuals are no longer looking younger biologically after each 3 cycle FMD and instead the decline due to FMD simply offsets the expected 1-year increase in biological age every year. This contributes to a slower rate of aging—biological age increases every year, but at a slower rate than chronological age. In comparison to the expected 1-year increase in biological age for every 1-year increase in chronological age, after stabilization, those undergoing 3 cycles of FMD annually exhibit only a 0.85-year increase in biological age for every 1 year of chronological age (Fig. S6A). Again, almost identical observation was found when the data from the second clinical trial data was used (Fig. S5E), as well as from the pooled samples (Fig. S5F).

This reduction in biological age is reflected in the extension of life expectancy estimates at age 70, assuming 3 FMD cycles annually starting at age 50 (Fig. S6B). While the estimated mean life expectancy differs by about 5 years, the predicted maximum life expectancy differs by 12 years. We then used this information to estimate the outcomes of either no FMD or consuming an FMD 3-times per year starting at age 50 (Fig. S6C). FMD estimates from the first clinical trial prolonged median expectancies by about 5 years in this model. For instance, median life expectancy at age 70 was 83.2 years for those not using the FMD; however, this increased to 88.3 years assuming continued FMD use. Similarly, the estimated 20-year risk of all-cause mortality at age 70 was reduced from 81.1% to 57.5% if FMD cycles were adopted. Estimates from the second trial indicate an increase in median life expectancies by 3.5 years (Fig. S5G) and pooled estimates indicate a 3.7-year increase (Fig. S5H).

Of note, the simulations discussed hereafter have limitations, including compliance, dropouts, biases that may arise from enthusiastic volunteers, and therefore the projections obtained by extrapolating the effects after 3–6 months of the FMD to a lifelong intervention have to be cautiously interpreted.

### Reductions in biological age by FMD may reduce risk for cause-specific mortality

Aging is associated with the increased risk of mortality from age-related diseases such as cardio-vascular disease, cancer, and diabetes. Previous studies have shown that biological age is a more robust estimate of these risks than chronological age. Using biological age estimates, we predicted what the changes in 20-year cause-specific mortality for all-causes, heart disease, cancer, cerebrovascular diseases, and diabetes might be—assuming changes in biological age are reflected in changing mortality risk. We found a reduction in mortality risk estimates after completion of the first FMD trial (Table S4). For instance, average 20-year all-cause mortality risk for trial study participants was predicted to be reduced from 11.25% to 10.07% according to changes in biological age following 3 cycles of FMD (10.5% reduction); mortality from heart disease was predicted to decline from 2.30% to 1.90%, representing a 17.4% reduction; 20-year cancer mortality risk was predicted at 4.54% at baseline and 4.25% at follow-up, representing a potential 6.4% reduction in risk; risk of mortality from cerebrovascular disease over 20 years was predicted to drop from 0.50% to 0.39%, signifying a 22% reduction; while diabetes mortality was estimated at 0.23% at baseline and 0.17% after 3 cycles of FMD, representing a 26% reduction. After adjusting our mortality risk models to assume a zero-pound weight loss, by adjusting the models for the change in biological age associated with the change in BMI, we still observe consistent decreases in the calculated mortality risk as a function of changes in biological age (Table S4). Notably, study participants with at least one elevated risk factor at baseline had increased 20-year cause-specific mortality risks for all-causes, heart disease, cancer, cerebrovascular diseases, and diabetes compared to all study participants at baseline and benefited most from the 3 FMD cycles as displayed in relative risk-reductions (Table S4).

Notably, these predictions could be invalidated by a catching up of the biological age markers with those of the untreated control populations years after the last FMD cycle. In fact, our previous study indicated a reduction in the effects of 3 FMD cycles already 3 months after the last cycle.

After simulating aging in the study participants of the first trial up to age 70 (chronologically), we estimated their cause-specific mortality risks based on models from NHANES III (used previously). This was done: (1) assuming participants did not undergo FMD and that at age 70, their Δ for the difference in biological and chronological age was consistent with what was observed at baseline and (2) assuming participants had performed 3 cycles of FMD annually (Fig. S6E–H). Based on the simulation, 20-year risk of mortality from heart disease was reduced from 29.1% to 15.4%; 20-year risk of cancer mortality was reduced from 26.0% to 18.9%; 20-year risk of mortality from cerebrovascular disease was reduced from 8.2% to 3.0%; and 20-year risk of diabetes-related mortality was reduced from 2.6% to 0.6%. Similar findings were, again, predicted based on estimates when simulating chronologically aging participants of the second trial, or pooled samples from both FMD trials, up to age 70 (Fig. S5G, H). If our assumptions are correct, these results suggest that yearly cycles of the FMD have the potential to cause changes in life expectancy, all-cause mortality and mortality caused by specific diseases.

## Discussion

Studies in humans indicate that the chronic reduction of calories 15–20% below the normal levels results in potent effects on risk factors for diabetes, cancer, and cardiovascular diseases. Studies in monkeys indicate that caloric restriction (CR) can prevent diabetes or insulin resistance in the great majority of the animals and cause major reductions in the incidence of both cancer and cardiovascular disease, whereas preliminary findings indicate that both CR and alternate-day fasting can lower insulin resistance and other risk factors for age related disease^52,53^. However, chronic CR is a severe intervention that would not be sustainable for the majority of the population but that also causes detrimental changes which might negate its beneficial effects- including a reduction in weight and lean body mass^54^. In fact, in one monkey study described above, decades of CR had strong effects on morbidity but causes a limited longevity extension, and in another it had no effects on longevity and minor effects on cancer prevention^55^. Thus, interventions that can match or surpass the beneficial effects of chronic CR on morbidity but associated with reduced burden, better compliance, and very low side effects are needed.

It is also necessary to develop methods that can allow the assessment of biological age, therefore avoiding the need to perform long clinical and epidemiological studies to determine healthspan and lifespan. The rate of aging is heterogeneous and thus chronological time (‘age’) is not a reliable proxy of the physiologically changes that are associated with the biological aging process^45^. Therefore, biological age measures, like the KDM biological age, which merge multi-system biomarkers into a single variable meant to capture the level and rate of organismal aging, are now validated in a number of studies^18–20,33,34^. Based on this measure, an individual’s biological age can be interpreted as the age in a representative population that his/her biological profile most closely corresponds to, given a set of clinical multi-system biomarkers. For instance, a person may have a chronological age of 50, but on a biological level, they may better resemble the average 55-year-old in the population, and thus have a mortality and morbidity risk more akin to that of a 55-year-old. Furthermore, because the markers which make-up the composite biological age are modifiable, this measure may facilitate evaluation of interventions, such as our FMD trial, aimed at slowing the aging process and delaying disability and disease.

In general, our cohort was made up of individuals who were healthier than the average person in the U.S. population, given that on average they were predicted to be 3.5 years younger biologically compared to their chronological ages at baseline. This likely represents an enrollment bias as education years and SES are higher in our sample compared to the average American population. This is a common phenomenon in clinical trials as volunteers are often motivated and more likely to be health conscious^56^. Regardless, even in this relatively healthy group, positive changes in the biomarkers used to estimate biological age led to a decrease in median biological age of 2.5 years following the completion of 3 FMD cycles. Even though at baseline the generally healthy volunteers in the FMD trial already had lower than average risks for heart-disease, diabetes and cerebrovascular disease, our simulations after 3 FMD cycles indicates biological age reductions associated with further decreases in the 20-year risk for all-cause and cause-specific mortality. It should be noted that these are estimated risk reductions and decreases in mortality were not directly observed in this study. It assumes that associations between biological age and mortality also reflect the effect of change in biological age—which has yet to be proven. However, these results provide preliminary evidence of the potential benefits of FMD even among healthy individuals.

Unhealthy dietary habits and the associated obesity pandemic have been linked to many diseases, including diabetes, CVD, certain cancers, and non-alcoholic fatty liver disease, and an accelerated rate of aging itself. In our first study on the FMD effects in a randomized cross-over trial, we reported that the efficacy of the FMD on clinically relevant risk factors was higher in at-risk participants than in those study participants with risk factor values within the normal range^34^. Here, we observed similar effects on biological age estimates: the volunteers who most benefitted from the FMD intervention where those who were most unhealthy at baseline. Estimates based off the effects of the FMD trial suggest that early deaths may be delayed whereas maximal life expectancy is probably not extended. For instance, estimated life expectancies in the highest ranges did not show much differences between pre- and post-intervention.

Here we begin to provide potential mechanisms that may explain the reduction of biological age by FMD cycles: in MRI volunteers the FMD cycles lowered total and visceral fat and lowered the hepatic fat fraction in study participants with overweight and in study participants with non-alcoholic fatty liver disease, and lower insulin resistance and HbA1c in a small subset of study participants which builds on our previous clinical and pre-clinical results to indicate that FMD cycles help prevent and can be considered as a therapy to prevent metabolic syndrome and diabetes^2,57^. When controlling for weight loss the effects persisted, implying that the improvements were probably affected by but were not a reflection of weight loss. Finally, the correlation between the changes in the individual biomarkers and the changes in biological age may suggest that a large proportion of these improvements resulted from shared mechanisms, such as general rejuvenating effects in cells and organs leading to reductions in systemic inflammation as indicated by the high correlations between changes in biological age and changes in both CRP and albumin. This possibility is also supported by the effect of the FMD cycles in reducing the age-associated shift in the lymphoid-to-myeloid ratio towards a younger phenotype, which matches well our previous results in model organisms^33^. A number of our mouse studies indicate that these effects of the FMD cycles are at least in part caused by multi-system effects involving a coordinated initial reduction in cell number and organ/system size, associated with an increase in stem cells number and proliferation as well as cellular reprogramming leading to a temporary embryonic-like gene expression profile followed by regeneration during the return to the normal size/cell number upon re-feeding^30–33^.

Based on the changes observed in the previous and current analyses of the data, our simulation suggests that prolonged practice of FMD or similar dietary interventions may lead to improvements in population health. For instance, we estimated that if individuals underwent 3 FMD cycles each year, they would decelerate their rate of aging, such that for every one chronological year, they would gain less than a year in biological age. A simulation of the effect of 3 FMD cycles for twenty years assuming continued efficacy of this dietary intervention suggests a potential decrease in biological age of about 11 years. Given that the risks for most major chronic diseases rise exponentially with biological age, the slowing of the aging process could lead to a prolonged disease-free life expectancy by delaying the onset of age-related chronic conditions or slowing the accumulation of multi-morbidities. For instance, it has been estimated that delaying the aging process by seven years could cut mortality from age-related diseases in half at nearly every age^58^. It was also recently estimated that a 20% slowing of the aging process could have an economic value of $7.1 trillion over the next 50 years^59^. Even under the assumption that the effect of FMD weakened each time it was performed, the results from our simulation suggest that prolonged practice of FMD may have the potential to slow the rate of aging, extend life expectancy, and cut the risks of disease specific mortality.

However, there are major limitations that need to be acknowledged in regards to our simulation: First, the models used to estimate the effects of 3 cycles of FMD on Biological age were based on results from two clinical trials and include only 86 participants. Moreover, trial participants generally shared advantageous social, economic, behavioral, and health characteristics and therefore their results may not generalize to the population as a whole. For instance, other characteristics that are not exemplified by our study participants may alter the effect of FMD on biological age, potentially leading to an overestimation of the long-term effects in our simulation. The simulation also does not take into account compliance, dropout, or the bias that may arise as a result of enthusiastic volunteers. For example, for a portion of the population it could be difficult to undergo 3 yearly FMD cycles for decades. Therefore, the effects are based on a compliance that may be feasible only for a portion of the population, although the clinical trials on normal, hypertensive, cancer, diabetes and Alzheimer patients indicate that even 6-12 consecutive monthly FMD cycles are feasible for the majority of the participants. Also, projections obtained by extrapolating the effects after 3–6 months of the FMD to a lifelong intervention should be cautiously interpreted, since they may be erased in study participants returning to their previous lifestyle in the absence of additional FMD cycles. Notably, although both trials included patients that were in the 60 s and reached 70 years of age and did not display age-dependent side effects, it has not been conclusively established whether the FMD may affect lean body mass and metabolic parameters in a harmful way or interfere with medications in the elderly, although both of the trials described in this study indicate no lean body mass loss after 3-4 consecutive monthly FMD cycles CITE 34 and 36. In fact, in the over 65-70 population, which begins to lose weight and lean body mass, 3 yearly FMD cycles may be as effective as suggested by our mouse study^33^ and our study on the effect of protein restriction in over 65 individuals^16^. However, results from an Alzheimer disease trial recently reported by our group with participants in the 55-80 age range indicated that the majority of subjects could complete an average of 6 FMD cycles without safety concerns, indicating that additional trials are necessary to determine whether FMDs may also be effective in the elderly 10.1016/j.celrep.2022.111417. We also did not account for the possibility of mortality in the simulation, and thus results are assuming all individuals remain alive.

It will be important to compare the FMD cycles with other dietary intervention studies in humans such as the CALERIE projects or the University of Illinois at Chicago’s alternate day modified fasting trial to estimate whether these interventions also affect biological age. Together our findings indicate that the FMD is a feasible periodic dietary intervention that reduces disease risk factors and biological age.

## Methods

### Study participants and study design (previously published)

Experimental design and report were prepared following the CONSORT standards for randomized clinical trials where applicable. The design and primary/secondary outcomes of the two human FMD trials have been reported for NCT02158897 in ref. ^34^ and for NCT04150159 in ref. ^36^. Both trials comply with all relevant ethical regulations and have been approved by the University of Southern California (USC) Institutional Review Board as well as the Institutional Review Board of the Hypertension Institute (HTI; Tennessee, United States).

Trial NCT02158897 (published before): one-hundred participants (generally healthy adult volunteers and 18 to 70 years of age; BMI, 18.5 and up) without a diagnosed medical condition in the previous 6 months were enrolled. Flow of participant enrollment and participation was prepared following the CONSORT standards for randomized clinical trials with crossover design. All participants provided written informed consent and the University of Southern California (USC) Institutional Review Board approved the protocol (HS-12-00391). Pre-specified outcome measures included safety and feasibility, and evaluation of changes in metabolic risk factors for diabetes and CVD and metabolic markers associated with age-related diseases and mortality; these outcomes were measured at baseline during and after completion of the intervention. All data were collected at the USC Diabetes and Obesity Research Institute. Study participants were recruited throughout Los Angeles County and surrounding areas from April 2013 to July 2015 based on established inclusion (generally healthy adult volunteers and 18 to 70 years of age; BMI, 18.5 and up) and exclusion [any major medical condition or chronic diseases, mental illness, drug dependency, hormone replacement therapy (dehydroepiandrosterone, estrogen, thyroid, and testosterone), pregnant or nursing female, special dietary requirements or food allergies, alcohol dependency, and medications known to affect body weight] criteria. Participants were not compensated. Hispanics (27%) were underrepresented in the study population in comparison to their representation (~45%) in the greater Los Angeles area (California, USA). Intention to treat analysis was performed by including all available observations. Eligible participants were randomly assigned using a random-number generator to either arm of the study. Participants were instructed to consume the FMD, which was provided in a box, for 5 continuous days, and to return to their normal diet after completion until the next cycle that was initiated about 25 days later. Participants completed three cycles of this 5-day FMD. Participants completed baseline (BL) and follow-up examinations at the end of the first FMD (before resuming normal diet to measure the acute FMD effects; 1 FMD) and after a washout period of 5 to 7 days of normal caloric intake after the third FMD cycle (3 FMD). Baseline characteristics before crossover for this study are as follows: - sex (self-reported) FMD cohort 19 male, 33 female (*N* = 52), normal diet cohort 18 male, 30 female (*N* = 48); - age FMD cohort 43.3 ± 11.7 vs. normal diet cohort 42.2 ± 12.5. In the randomized comparison, 18 participants (5 of 48 (10%) in the control arm and 13 of 52 participants in the FMD arm (25%)) were excluded or withdrew from the study. In the control arm, two withdrew because of scheduling conflicts, two because of unspecified personal issues, and one for unknown reasons. 6 of the 52 study participants enrolled in the FMD arm withdrew from the study because of scheduling conflicts, five withdrew because of unspecified personal issues, and two participants were excluded from the study because of noncompliance to the FMD protocol.

Trial NCT04150159 (published before): This trial was designed as a single center parallel-group clinical trial to evaluate the effects of the FMD in comparison with a Mediterranean diet and was approved by the Institutional Review Board of the Hypertension Institute (HTI; Tennessee, United States) where participants were recruited between November 2019 and December 2019. All participants provided written informed consent. Participants were not compensated. Participants were randomized to the FMD or to a Mediterranean Diet arm using a randomization schedule in blocks of four stratified by sex. Study participants randomized to the FMD were instructed to consume the FMD for 5 continuous days, and to return to their normal diet after completion until the next cycle that was initiated about 25 days later for a total of 4 FMD cycles. Eligible participants were men and women with (i) BMI ≥ 28, (ii) diagnosed with either endothelial dysfunction (based on Reactive hyperemia index RHI ≤ 2.0), or (iii) low small resistance artery compliance (AC2 ≤ 5.0). Exclusion criteria included being pregnant or lactating, allergies to nuts or other study products, excessive consumption of alcoholic beverages, drug abuse, clinically significant vital signs abnormalities (systolic blood pressure <90 mm Hg or >180 mm Hg, diastolic blood pressure <50 mm Hg or >105 mm Hg, at screening), serious unstable illness, any known serious infection (HIV, TB, hepatitis B or C), current diagnosis or personal history of major cardiometabolic disorders, or serious mental illness. Baseline characteristics for this study are as follows: - sex (self-reported) FMD cohort 12 male, 32 female (*N* = 44), MD cohort 20 male, 20 female (*N* = 40); - age FMD cohort 54.9 ± 12.4 vs. MD cohort 63.1 ± 7.72.

### Experimental fasting-mimicking diet (previously published)

The FMD is a plant-based diet designed to attain fasting-like effects on the serum levels of IGF-1, IGFBP-1, glucose, and ketone bodies while providing both macro- and micronutrients to minimize the burden of fasting and adverse effects. Day 1 of the FMD supplies ~4600 kJ (11% protein, 46% fat, and 43% carbohydrate), whereas days 2 to 5 provide ~3000 kJ (9% protein, 44% fat, and 47% carbohydrate) per day. The FMD provided by L-Nutra to this study participants comprises proprietary formulations belonging to USC and L-Nutra (www.prolonfmd.com) of vegetable-based soups, energy bars, energy drinks, chip snacks, tea, and a supplement providing high levels of minerals, vitamins, and essential fatty acids^34^. All items to be consumed per day were individually boxed (Prolon) to allow the study participants to choose when to eat while avoiding accidentally consuming components of the following day.

### Hepatic Fat Fraction (reported here)

Magnetic resonance imaging (MRI) was used to measure hepatic proton density fat fraction (PDFF). Approximately 50 5 mm axial slices were obtained from the top of the liver to the pelvis using a series of 10–15 s breath holds. Imaging was performed on a GE Signa EXCITE HDxt 3.0 T MR scanner using the iterative decomposition of water and fat with echo asymmetry and least-squares estimation (IDEAL) method with T2* and fat spectrum modeling^60,61^. The accuracy and precision of in-vivo hepatic PDFF measurements using this approach has been established in previous studies^62^.

### Blood tests and serum markers (reported here)

Complete metabolic and lipid panels (overnight fasting) were completed at the Clinical Laboratories at the Keck Medical Center of USC and analyzed immediately after the blood draw of each visit. Commercially available kits for CRP (R&D Systems DCRP00) and β-hydroxybutyrate (Cayman Chemicals 700190) were used following the manufacturers’ protocols. Plasma IGF-I and IGFBP-1 levels were analyzed by an in-house ELISA.

### Biological age estimation (reported here)

Biological age was estimated based on information on seven clinical chemistry measurements (Table S1) using the method provided in refs. ^45,63^. In short, the estimated Biological Age calculation combines information from *m* = 7 regression lines of chronological age regressed on *m* = *7* biomarker indicators (Eq. 1).

1 $${{BA}}_{{EC}}=\frac{\mathop{\sum }\nolimits_{j=1}^{m}({x}_{j}-{q}_{j})\frac{{k}_{j}}{{s}_{j}^{2}}+\frac{{CA}}{{s}_{{BA}}^{2}}}{\mathop{\sum }\nolimits_{j=1}^{m}{\left(\frac{{k}_{j}}{{s}_{j}}\right)}^{2}+\frac{1}{{s}_{{BA}}^{2}}}$$

The above parameters used in the biological age equation were calculated based on 10,519 participants ages 30–90 from the Third National Health and Nutrition Examination Survey (NHANES III). In Equation 1, *k*_*j*_ and *q*_*j*_ represent the slope and intercept, respectively, for chronological age regressed on each biomarker; *x*_*j*_ represents a participant’s measured value for the given biomarker; *s*_*j*_ represents the root mean squared error of chronological age regressed on a biomarker, and *CA* represents chronological age. Additionally, ${s}_{{BA}}^{2}$ is the variance of the random variable, *R*_*BA*_, which represents the mean difference between participants’ biological and chronological age. To calculate the biological ages of the clinical trial study participants at three time-points, we combined the *k*_*j*_, *s*_*j*_, *q*_*j*_, and$\,{s}_{{BA}}^{2}$ estimated in NHANES III with the study participants chronological age (CA) and his/her levels for each biomarker (*x*_*j*_).

For the BMI adjusted biological ages, we fit a linear model for changes in BMI regressed on changes in biological age and then used this to calculate the residual of changes in biological age for each study participants as a function of his/her change in BMI. By assessing the association between change in weight and follow-up biological age, we calculated a weight-adjusted biological age measure based on the residual. We regressed follow-up biological age on the change in weight (weight follow-up minus weight baseline). Using the equation from this model, we estimated residuals for follow-up biological age. Overall, this value represents what the follow-up biological age would be estimated to be assuming no change in weight had occurred. Regardless, we estimated the association between change in weight and change in biological age and then calculated the weight adjusted biological ages at follow-up—biological age assuming no change in weight. To do this, change in biological age was regressed on change in weight, and the residual from this was used to assess the impact of FMD on biological age, independent of the effect from change in weight.

### Life expectancy and mortality risk estimations (reported here)

Up to 23 years of mortality follow-up was available for NHANES III, as provided via linked mortality files taken from the National Death Index through 2011. Life expectancy was estimated based on parameters calculate in NHANES III from a proportional hazard model with Gompertz distribution, in which time-to-death was regressed on biological and chronological age (Eqs. 2 and 3). Remaining median life expectancy is estimated by solving for $t$ when $s\left(t\right)=0.50$—representing the length of time until 50% of the population with a given biological and chronological age has died/survived.

2 $${\lambda }_{j}=\exp \left({{Age}}_{j}{\beta }_{{Age}}+{{BioAge}}_{j}{\beta }_{{BioAge}}+{const}.\right)$$

3 $$s\left(t\right)=\exp \left\{\left(\frac{{\lambda }_{j}}{\gamma }\right)\left({1-e}^{\gamma t}\right)\right\}$$

Similarly, 20-year mortality risk estimates were calculated by solving for *s(t)* in Eq. 3, in which *t* is set equal to 20 years. For these estimates, separate competing-risk models were fit for each cause-specific mortality outcome and then incorporated into Eqs. 2 and 3. Finally, weight adjusted models for both life expectancy and mortality risk combined Eq. 4 (includes weight) and Eq. 3. When the parameters were applied to the clinical trial data, weight at baseline was included in both the baseline and post-intervention models to simulate changes in life expectancy and mortality risk assuming no change in weight.

4 $${\lambda }_{j}=\exp \left({{Age}}_{j}{\beta }_{{Age}}+{{BioAge}}_{j}{\beta }_{{BioAge}}+{{Weight}}_{j}{\beta }_{{Weight}}+{const}.\right)$$

### Statistical analysis(reported here)

For this randomized trial (NCT02158897), the sample size of 100 total study participants was based on the detection of a 25% reduction in mean IGF-1, with a two-sided α of 0.05 and 70% power. The estimated control group mean (SD) IGF-1 of 194 (97) used published data on males and females aged 26 to 40 years^35^.

Wilcoxon Rank Sum Scores were used to assess for changes in biological age following 3 cycles of FMD. For this analysis, three models were run—one for the full sample, one using the biological age estimate adjusted for weight (excluding the effect of changes in weight), and one including study participants with at least one risk factor. To examine what baseline factors were associated with response to FMD, we generated a binary variable that denoted whether participants exhibited decreased biological age following FMD (denoted as 0) or increased biological age following FMD (denoted as 1). Multivariate logistic regression was then run to test the association between baseline characteristics and the odds of being in the non-response group. Next, we estimated the median life expectancies and 20-year mortality risks comparing baseline and follow-up. Given that these estimates are both direct transformations of the biological age measure there was no need to re-test for significant changes as a function of the FMD trial—results would mirror what was found for biological age, since those who show decreases in biological age also show increases in life expectancy and decreases in mortality risk. Biweight midcorrelation was used to test for an association between weight loss and changes in biological age and the individual biomarkers following the FMD. Biweight midcorrelation is similar to a Pearson correlation, while also accounting for outliers^64^.

### Simulation (reported here)

A simulation of the effect of continued FMD (3 cycles annually up until age 70) was run using the NHANES IV population ages 20–59 (*n* = 7733), given that it is the closest representation of the current U.S. population. Two OLS models were estimated from the clinical trial data. The first model represented the effect of baseline chronological and biological age on the change in biological age following 3 cycles of FMD over 3 months (Eq. 5). The second model (Eq. 6) represented the rebound—or change in BioAge after returning to normal diet for 3 months following the FMD.

5 $${{Change}\,{BioAge}}_{A\,{to}\,C}=0.1017-\left(0.1906 \,\ast\, {BioAge}\right)+(0.1227\, \ast\, {Age})$$

6 $${{Change}\,{BioAge}}_{C\,{to}\,D}=0.6687-\left(0.5567 \,\ast\, {{BioAge}}_{A}\right)+\left(0.4008 \,\ast\, {{BioAge}}_{C}\right)+(0.1157 \,\ast\, {Age})$$

The simulation for model 1, followed by model 2 was repeated to simulate the set regression coefficients and standard errors with uncertainty. Sample weights were used to infer how many simulations to run when calculating means. For instance, the NHNAES IV fasting weight was divided by 548.5 and rounded to the nearest whole number, so that it ranged from 6 to 1000 (mean = 207, median 144). If a participant had a sampling weight of six, we selected six of the 1000 simulations, for which the mean was used to represent the predicted value for that individual. The simulation was carried out over a hypothetical period that lasted from the time at baseline to when each individual would have reached 70 years of age. For each subsequent year after baseline, the BioAge a study participants was predicted to reach at the end of the prior year was now used as input into Eq. 5. For instance, an individual from NHANES who was 40 years old chronologically and estimated to have a BioAge of 45 at baseline, may have been predicted to decrease in BioAge to 43. If that were the case, the simulation for the following year would start with a chronological age of 41 (40 + 1) and BioAge of 43 to predict another change after 3 cycles of FMD and a rebound period. This would continue for 30 years (until the person reached 70 years of chronological age).

As a sensitivity analysis, we repeated the entire simulation, but for each year following baseline, we reduced the effect size for change in BioAge by dividing by 2 multiplied by years since baseline. This represented a reduction in the effect of FMD with each subsequent year, such that in year 2 it was half as effective as year one, and in year 3 it was a quarter as effective, and so on and so forth.

### Reporting summary

Further information on research design is available in the Nature Portfolio Reporting Summary linked to this article.

### Supplementary information

Supplementary Information

Reporting Summary

### Source data

Source Data

## Data Availability

The datasets supporting the findings described in this manuscript are available in the article and in the Supplementary Information and can be obtained from the corresponding author upon request. Source data are provided with this paper^65^. Source data are provided with this paper.

## Code availability

The codes used in the current study are described in this manuscript and available from the corresponding authors on request. Source codes are provided with this paper^66^.
