# Supplementary material for: Fasting-mimicking diet causes hepatic and blood markers changes indicating reduced biological age and disease risk
Source: Nat Commun. 2024 Feb 20;15:1309. doi: 10.1038/s41467-024-45260-9 (PMC10879164; doi:10.1038/s41467-024-45260-9)
Supplement: Supplementary file 1 — Supplementary Information [file 41467_2024_45260_MOESM1_ESM.pdf]

Fasting-mimicking diet causes hepatic and blood markers changes indicating reduced biological age and disease risk

Supplementary Data

Supplementary Table 1 Arm-specific markers of adherence and changes in risk factors of NCT02158897

Variable	N=	Baseline<br>Mean ± SD	**CTRL:** 3 months after Baseline<br>**FMD:** 5 Days After 3rd FMD Cycle<br>Mean ± SD	p-value†	Δ*	Efficacy p-value[§] (**FMD** Arm 2 *vs.*)<br>**CTRL** (Arm 1)	**FMD** (Arm 1)	Power
**Body Weight** (kg)								
**Control Diet**, Arm 1	*43*	77.2 ± 16.5	77.3 ± 17.0	0.69	0.1 ± 2.1	< 0.0001		0.06
**FMD**, Arm 2	*39*	74.1 ± 15.5	71.6 ± 14.6	< 0.0001	-2.6 ± 2.5		0.12	1.00
**FMD**, Arm 1	*32*	79.0 ± 18.4	77.3 ± 17.5	< 0.0001	-1.7 ± 2.0			1.00
**FMD**, Arm 1 and 2	*71*	76.3 ± 16.9	74.1 ± 16.1	< 0.0001	-2.2 ± 2.3			1.00
**Body-mass Index‡**								
**Control Diet**, Arm 1	*43*	27.4 ± 4.8	27.4 ± 5.0	0.90	0.0 ± 0.7	< 0.0001		0.05
**FMD**, Arm 2	*39*	26.2 ± 4.4	25.3 ± 4.3	< 0.0001	-0.9 ± 0.9		0.07	1.00
**FMD**, Arm 1	*32*	27.4 ± 5.3	26.8 ± 5.1	< 0.0001	-0.6 ± 0.7			1.00
**FMD**, Arm 1 and 2	*71*	26.7 ± 4.8	26.0 ± 4.5	< 0.0001	-0.7 ± 0.8			1.00
**Total Body Fat**[ɣ] (abs. Volume)								
**Control Diet**, Arm 1	*43*	23651 ± 8155	23607 ± 8337	0.54	-44 ± 1365	< 0.001		0.08
**FMD**, Arm 2	*38*	20643 ± 8459	19249 ± 7792	< 0.001	-1393 ± 1786		0.67	1.00
**FMD**, Arm 1	*32*	22841 ± 8733	21618 ± 8567	< 0.001	-1222 ± 1484			0.99
**FMD**, Arm 1 and 2	*70*	21648 ± 8594	20332 ± 8182	< 0.0001	-1315 ± 1646			1.00
**Total Body Fat**[ɣ] (rel. Volume %)								
**Control Diet**, Arm 1	*43*	33.4 ± 8.6	33.3 ± 9.0	0.57	-0.1 ± 1.5	< 0.05		0.07
**FMD**, Arm 2	*38*	30.3 ± 10.0	29.4 ± 9.8	< 0.01	-0.9 ± 2.0		0.72	0.77
**FMD**, Arm 1	*32*	31.4 ± 8.9	30.3 ± 8.8	< 0.01	-1.1 ± 1.8			0.97
**FMD**, Arm 1 and 2	*70*	30.8 ± 9.5	29.8 ± 9.3	< 0.001	-1.0 ± 1.9			0.99
**Trunk Fat**[ɣ] (abs. Volume)								
**Control Diet**, Arm 1	*43*	8429 ± 4742	8395 ± 4776	0.51	-33 ± 1046	< 0.05		0.08
**FMD**, Arm 2	*38*	6573 ± 4877	5938 ± 4295	< 0.01	-636 ± 1198		0.77	0.89
**FMD**, Arm 1	*32*	7807 ± 4848	7092 ± 4578	< 0.001	-715 ± 1015			0.97
**FMD**, Arm 1 and 2	*70*	7137 ± 4869	6465 ± 4432	< 0.0001	-672 ± 1111			0.89
**Trunk Fat**[ɣ] (rel. Volume %)								
**Control Diet**, Arm 1	*43*	11.7 ± 5.7	11.7 ± 6.0	0.43	0.0 ± 1.3	0.07		0.05
**FMD**, Arm 2	*38*	9.5 ± 6.1	8.9 ± 5.6	< 0.01	-0.6 ± 1.4		0.47	0.73
**FMD**, Arm 1	*32*	10.5 ± 5.9	9.7 ± 5.4	< 0.01	-0.8 ± 1.5			0.83
**FMD**, Arm 1 and 2	*70*	9.9 ± 6.0	9.2 ± 5.5	< 0.001	-0.7 ± 1.4			0.98
**Lean Body Mass**[ɣ] (abs. Volume)								
**Control Diet**, Arm 1	*43*	45016 ± 11318	45188 ± 11885	0.59	172 ± 1441	< 0.001		0.19
**FMD**, Arm 2	*38*	45321 ± 11794	44231 ± 11247	< 0.001	-1089 ± 1613		0.08	0.98
**FMD**, Arm 1	*32*	47451 ± 12323	47016 ± 11555	0.15	-411 ± 1575			0.78
**FMD**, Arm 1 and 2	*70*	46281 ± 11997	45501 ± 11392	< 0.0001	-780 ± 1620			0.99
**Lean Body Mass**[ɣ] (rel. Volume %)								
**Control Diet**, Arm 1	*43*	63.9 ± 8.2	64.0 ± 8.7	0.57	0.1 ± 1.5	0.07		0.07
**FMD**, Arm 2	*38*	66.8 ± 9.6	67.6 ± 9.4	< 0.05	0.8 ± 2.0		0.67	0.67
**FMD**, Arm 1	*32*	65.8 ± 8.6	66.8 ± 8.4	< 0.01	1.0 ± 1.8			0.86
**FMD**, Arm 1 and 2	*70*	66.3 ± 9.1	67.2 ± 9.2	< 0.001	0.9 ± 1.9			0.97
**Waist Circumference** (cm)								
**Control Diet**, Arm 1	*28*	95.4 ± 14.2	94.6 ± 14.5	0.13	-0.8 ± 25	< 0.01		0.05
**FMD**, Arm 2	*28*	92.1 ± 11.2	87.9 ± 120	< 0.001	-4.1 ± 5.2		0.07	0.98
**FMD**, Arm 1	*24*	97.6 ± 15.5	95.8 ± 13.7	< 0.05	-1.8 ± 3.6			0.65
**FMD**, Arm 1 and 2	*52*	94.6 ± 13.5	91.6 ± 13.3	< 0.0001	-3.0 ± 4.6			1.00

Variable	N=	Baseline<br>Mean ± SD	CTRL: 3 months after Baseline<br>FMD: 5 Days After 3rd FMD Cycle<br>Mean ± SD	p-value†	Δ*	Efficacy<br>p-value[§] (**FMD** Arm 2 *vs.*)<br>**CTRL** (Arm 1)	**FMD** (Arm 1)	Power
**Fasting Glucose** (mg/dL)								
**Control Diet**, Arm 1	*41*	88.1 ± 8.9	90.3 ± 9.7	0.15	2.2 ± 9.5	0.26		0.30
**FMD**, Arm 2	*36*	89.7 ± 8.5	89.0 ± 8.0	0.65	-0.8 ± 9.9			0.08
**FMD**, Arm 1	*30*	93.0 ± 9.1	90.2 ± 8.4	0.09	-2.8 ± 8.8		0.39	0.39
**FMD**, Arm 1 and 2	*66*	91.0 ± 9.0	89.7 ± 8.2	0.41	-1.3 ± 9.8			0.19
**Fasting Insulin** (uIU/mL)								
**Control Diet**, Arm 1	*26*	6.7 ± 3.7	5.5 ± 3.8	< 0.05	-1.2 ± 2.0	0.08		0.84
**FMD**, Arm 2	*23*	5.7 ± 4.4	5.8 ± 5.3	0.88	0.1 ± 2.8			0.05
**FMD**, Arm 1	*22*	5.7 ± 3.8	5.1 ± 5.1	0.42	-0.6 ± 3.2		0.47	0.13
**FMD**, Arm 1 and 2	*45*	5.7 ± 4.1	5.5 ± 5.2	0.20	-0.2 ± 3.0			0.07
**HOMA-IR** (rel. Units)								
**Control Diet**, Arm 1	*25*	1.5 ± 0.9	1.3 ± 0.9	< 0.05	-0.2 ± 0.5	0.15		0.48
**FMD**, Arm 2	*23*	1.3 ± 1.1	1.3 ± 1.4	0.98	0.0 ± 0.7			0.05
**FMD**, Arm 1	*22*	1.3 ± 0.9	1.2 ± 1.3	0.51	-0.1 ± 0.8		0.59	0.09
**FMD**, Arm 1 and 2	*45*	1.3 ± 1.0	1.3 ± 1.3	0.14	0.0 ± 0.8			0.05
**β-Hydroxybutyrate** (mM)								
**Control Diet**, Arm 1	*42*	0.5 ± 0.4	0.5 ± 0.6	0.07	0.0 ± 0.8	0.82		0.05
**FMD**, Arm 2	*38*	0.4 ± 0.3	0.4 ± 0.3	0.55	0.0 ± 0.3			0.05
**FMD**, Arm 1	*31*	0.4 ± 0.3	0.4 ± 0.2	0.29	0.0 ± 0.3		0.67	0.05
**FMD**, Arm 1 and 2	*69*	0.4 ± 0.3	0.4 ± 0.2	0.63	0.0 ± 0.3			0.05
**IGF-1** (ng/mL)								
**Control Diet**, Arm 1	*41*	180.2 ± 84.5	188.9 ± 91.0	0.32	8.7 ± 36.9	< 0.01		0.31
**FMD**, Arm 2	*38*	168.6 ± 69.1	146.9 ± 62.3	< 0.01	-21.7 ± 46.2			0.80
**FMD**, Arm 1	*31*	191.3 ± 75	162.9 ± 71.0	< 0.001	-28.4 ± 43.6		0.54	0.94
**FMD**, Arm 1 and 2	*69*	178.8 ± 72.2	154.1 ± 66.4	< 0.0001	-24.7 ± 44.9			0.99
**IGFBP-1** (ng/mL)								
**Control Diet**, Arm 1	*41*	35.5 ± 43.6	36.5 ± 47.9	0.77	1.0 ± 26.9	0.53		0.06
**FMD**, Arm 2	*38*	24.2 ± 17.1	22.2 ± 16.4	0.35	-2.0 ± 13.0			0.15
**FMD**, Arm 1	*31*	33.8 ± 51.2	33.9 ± 42.7	0.99	0.1 ± 62.2		0.84	0.05
**FMD**, Arm 1 and 2	*69*	28.5 ± 36.6	27.5 ± 31.4	0.55	-1.0 ± 42.4			0.05
**Systolic Blood Pressure** (mmHg)								
**Control Diet**, Arm 1	*43*	116.5 ± 12.3	115.8 ± 13.6	0.48	-0.7 ± 8.4	< 0.05		0.08
**FMD**, Arm 2	*38*	118.0 ± 13.4	113.5 ± 13.2	< 0.001	-4.5 ± 6.0			0.99
**FMD**, Arm 1	*32*	116.5 ± 12.6	113.6 ± 11.5	< 0.05	-2.9 ± 7.6		0.33	0.55
**FMD**, Arm 1 and 2	*70*	117.4 ± 13.0	113.6 ± 12.4	< 0.0001	-3.8 ± 6.8			1.00
**Diastolic Blood Pressure** (mmHg)								
**Control Diet**, Arm 1	*43*	75.5 ± 9.6	74.8 ± 10.0	0.45	-0.7 ± 6.2	0.05		0.11
**FMD**, Arm 2	*38*	75.7 ± 8.0	72.6 ± 8.7	< 0.001	-3.1± 4.7			0.98
**FMD**, Arm 1	*32*	75.6 ± 9.8	73.1 ± 9.0	< 0.05	-2.6 ± 5.9		0.65	0.68
**FMD**, Arm 1 and 2	*70*	75.7 ± 8.8	72.8 ± 8.8	< 0.001	-2.9 ± 5.3			0.99

Variable	N=	Baseline<br>Mean ± SD	CTRL: 3 months after Baseline<br>FMD: 5 Days After 3rd FMD Cycle<br>Mean ± SD	p-value†	Δ*	Efficacy p-value[§] (FMD Arm 2 *vs.*)<br>CTRL (Arm 1)	FMD (Arm 1)	Power
**Triglycerides** (mg/dL)								
**Control Diet**, Arm 1	*37*	100.5 ± 68.2	101.5 ± 57.1	0.51	1.0 ± 35.0	0.27		0.05
**FMD**, Arm 2	*30*	83.0 ± 39.5	74.9 ± 37.6	0.19	-8.1 ± 33.5		0.86	0.26
**FMD**, Arm 1	*25*	114.6 ± 56.4	108.3 ± 51.8	0.48	-6.2 ± 43.4			0.11
**FMD**, Arm 1 and 2	*55*	97.4 ± 50.4	90.2 ± 47.5	0.10	-7.2 ± 38.2			0.28
**Total Cholesterol** (mg/dL)								
**Control Diet**, Arm 1	*37*	195.9 ± 38.9	183.9 ± 35.2	< 0.01	-12.0 ± 21.3	0.81		0.92
**FMD**, Arm 2	*30*	175.3 ± 25.3	164.4 ± 23.4	< 0.001	-10.9 ± 17.0		0.73	0.93
**FMD**, Arm 1	*25*	199.3 ± 35.2	190.2 ± 29.8	0.05	-9.1 ± 22.3			0.50
**FMD**, Arm 1 and 2	*55*	186.1 ± 32.5	176.2 ± 29.4	< 0.001	-9.9 ± 19.5			0.96
**LDL Cholesterol** (mg/dL)								
**Control Diet**, Arm 1	*37*	111.2 ± 35.6	104.0 ± 31.8	< 0.05	-7.2 ± 17.7	0.50		0.67
**FMD**, Arm 2	*30*	94.1 ± 23.0	89.7 ± 22.8	0.14	-4.4 ± 16.0		0.46	0.32
**FMD**, Arm 1	*25*	117.8 ± 36.3	110.1 ± 29.0	< 0.05	-7.8 ± 17.8			0.56
**FMD**, Arm 1 and 2	*55*	104.9 ± 32.0	99.2 ± 27.6	< 0.05	-5.7 ± 16.8			0.70
**HDL Cholesterol** (mg/dL)								
**Control Diet**, Arm 1	*37*	64.3 ± 16.1	59.3 ± 14.9	< 0.001	-5.3 ± 7.8	0.90		0.98
**FMD**, Arm 2	*30*	64.8 ± 17.2	59.6 ± 12.8	< 0.01	-5.0 ± 10.0		0.03	0.77
**FMD**, Arm 1	*25*	58.4 ± 16.2	58.4 ± 15.6	0.97	0.0 ± 5.8			0.05
**FMD**, Arm 1 and 2	*55*	61.7 ± 16.9	58.9 ± 14.1	0.07	-2.8 ± 8.7			0.65
**C-reactive Protein** (mg/L)								
**Control Diet**, Arm 1	*42*	1.5 ± 1.9	1.9 ± 2.7	0.48	0.4 ± 2.5	0.27		0.17
**FMD**, Arm 2	*38*	1.1 ± 1.3	1.0 ± 1.2	0.61	-0.1 ± 1.5		0.10	0.07
**FMD**, Arm 1	*31*	1.9 ± 3.0	0.9 ± 1.2	0.05	-1.0 ± 2.8			0.49
**FMD**, Arm 1 and 2	*69*	1.5 ± 2.2	1.0 ± 1.2	< 0.05	-0.5 ± 2.2			0.46
**Glucose Tolerance Test** (AUC)								
**FMD**, Glucose	*21*	16002 ± 2891	16599 ± 2381	0.08	592 ± 1870			0.28
**FMD**, Insulin	*11*	3704 ± 1210	5152 ± 1986	< 0.05	1448 ± 1526			0.81
**MSPC** (% mono-nucleated cell population)								
**Control Diet**, Arm 1	*21*	0.5 ± 1.0	0.6 ± 2.0	0.47	0.1 ± 1.6			0.06
**FMD**, Arm 1 and 2 AC	*34*	0.2 ± 0.5	0.7 ± 1.7	0.05	0.5 ± 1.8			0.35

** Plus-minus values are mean± SD rounded to the nearest tenth.*

† p-values for differences were calculated by two-tailed Wilcoxon matched pair tests with 95% Confidence Intervals compared to baseline measurements (A) and considered significant if p< 0.05.

§ p-values for differences were calculated by two-tailed Student's t-test with 95% Confidence Intervals between the Δ changes of each subject and parameter and considered significant if p< 0.05.

‡ The body-mass index is the weight in kilograms divided by the square of the height in meters.

¥ Analysed by dual energy x-ray absorptiometry.

From Wei, Min et al. "Fasting-mimicking diet and markers/risk factors for aging, diabetes, cancer, and cardiovascular disease." Science translational medicine vol. 9,377 (2017): eaai8700. doi:10.1126/scitranslmed.aai8700. Reprinted with permission from AAAS.

Supplementary Table 2: Parameters for Estimation of Levine Biological Age

Biomarker	Units	sj	kj	qj
Albumin	g/dL	0.334481	-0.00544	4.423451
Alkaline Phosphatase	u/L	29.44492	0.443863	60.44123
Creatinine (Serum)	mg/dL	0.271155	0.003463	0.908423
CRP	mg/dL	0.615577	0.004941	0.179063
Hba1c	%	0.943548	0.017761	4.5679
Systolic BP	mmHg	14.94318	0.677014	90.99925
Total Cholesterol	mg/dL	40.3238	0.793224	170.8787

Biological age was estimated based on information on seven clinical chemistry measurements.

Supplementary Table 3: Wilcoxon Rank Sum Scores for Change in Biological Age Between Baseline and the 3rd FMD cycle.

		N	Sum Ranks	Expected
**Complete Sample**	Decrease	36	1060	689
	Increase	16	318	689
	Unadjusted Variance=12,057.5<br>Z=3.38<br>P=0.0007			
**Adjusting for Change in BMI**	Decrease	31	895	663
	Increase	20	431	663
	Unadjusted Variance=11,381.5<br>Z=2.18<br>P=0.029			
**At least 1 Risk Factor**	Decrease	26	436	232.5
	Increase	4	29	232.5
	Unadjusted Variance=2,363.75<br>Z=4.19<br>P=2.79E-5			

<u>Complete sample</u>: After 3 FMD cycles, median biological age in the 52 study participants decreases by nearly 2.5 years. <u>Adjusting for change in BMI</u>: We estimate the association between change in BMI and change in biological age and then calculated the BMI adjusted biological ages at follow-up—biological age assuming no change in BMI. <u>At least 1 risk factor</u>: We restricted the sample to at-risk study participants (defined as having at least one of the following: BMI >25, elevated CRP, fasting glucose >99, and systolic hypertension) and re-evaluated the changes in biological age. sj represents the root mean squared error of chronological age regressed on a biomarker, kj and qj represent the slope and intercept, respectively, for chronological age regressed on each biomarker. Unadjusted two-sided p-values.

Supplementary Table 4. Risk estimates based on Gompertz proportional hazard model parameters calculated in NHANES III.

	**Normal Model** (N=51)			**Adjusting for BMI** (N=51)			**≥ 1 risk factor** (N=30)		
	**BL**	**3 FMD**	**Relative Reduction (%)**	**BL**	**3 FMD**	**Relative Reduction (%)**	**BL**	**FMD**	**Relative Reduction (%)**
**All Cause Risk**	11.25	10.07	10.49	11.38	10.18	10.54	15.4	13.3	13.64
**Heart Risk**	2.30	1.90	17.39	2.35	1.96	16.60	3.4	2.7	20.59
**Cancer Risk**	4.54	4.25	6.39	4.60	4.31	6.30	5.9	5.3	10.17
**Cerebrovascular Risk**	0.50	0.39	22.00	0.49	0.38	22.45	0.77	0.58	24.68
**Diabetes Risk**	0.23	0.17	26.09	0.24	0.18	25.00	0.34	0.23	32.35

Model 1: (Normal Model) included Levine Biological Age and chronological age as independent variables.
Model 2: (Adjusting for BMI) included variables from model 1, with the addition of a continuous BMI measure. Based on this model, mortality risks for clinical trial participants were estimated using their baseline BMI— signifying mortality risk assuming no change in BMI.
Model 3: (Adjusting for BMI) included Levine Biological Age and chronological age as independent variables for those study participants with at least one elevated risk factor.

Supplementary Figure 1: Associations between baseline characteristics and the probability of being a non-responder.

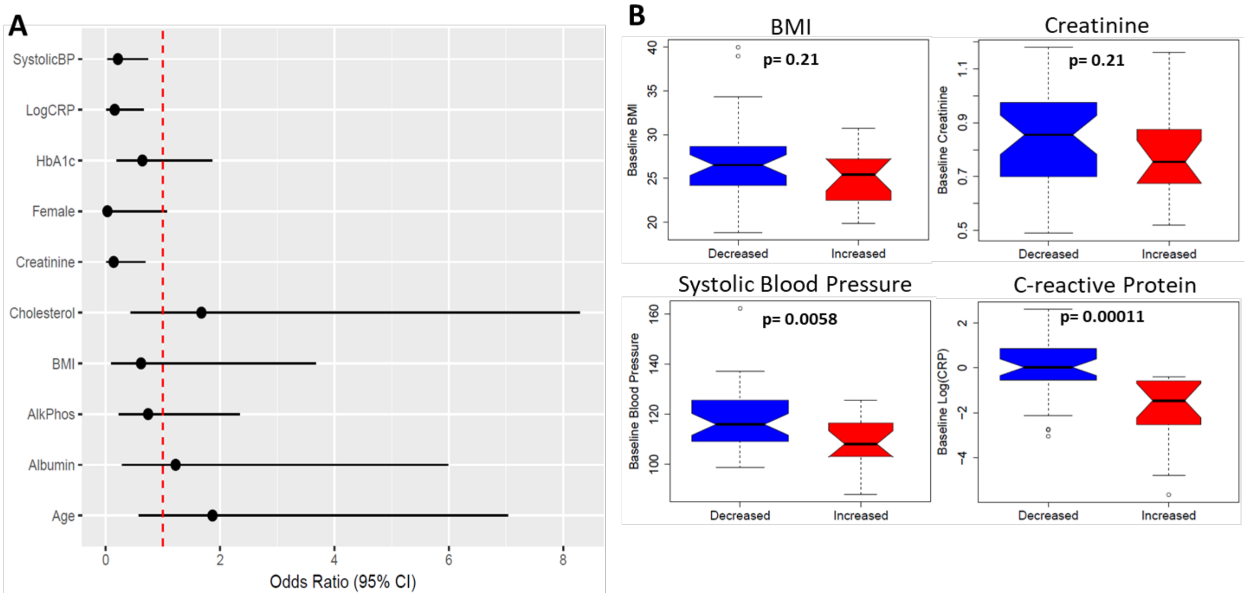

A) Forrest Plot of Baseline Characteristics and Response to FMD: study participants were coded as 1 if they exhibited increased biological age following FMD (non-responders), or 0 if they exhibited decreases in biological age following FMD (responders). Multivariate logistics regression was used to assess the association between baseline characteristics and the odds of being a non-responder. N=52 biologically independent samples. Dots represent odds ratios and error bars represent 95% confidence intervals. The red dotted line denotes an odds ratio of 1, suggesting no association. **B)** Boxplot of Select Baseline Variables Based on FMD Response: Descriptive statistics for baseline BMI, systolic blood treasure, CRP (log-transformed), and creatinine for responders (blue) and non-responders (red) were plotted. N=51 biologically independent samples. Center line = median, box bounds= interquartile range (IQR, 25th and 75th percentile), whiskers are 1.5xIQR, width= proportional to the square-roots of the number of observations if 'TRUE'. Kruskal-Wallis test was used and significance was assessed via unadjusted two-sided p-value.

Supplementary Figure 2: Individual biological-chronological age changes.

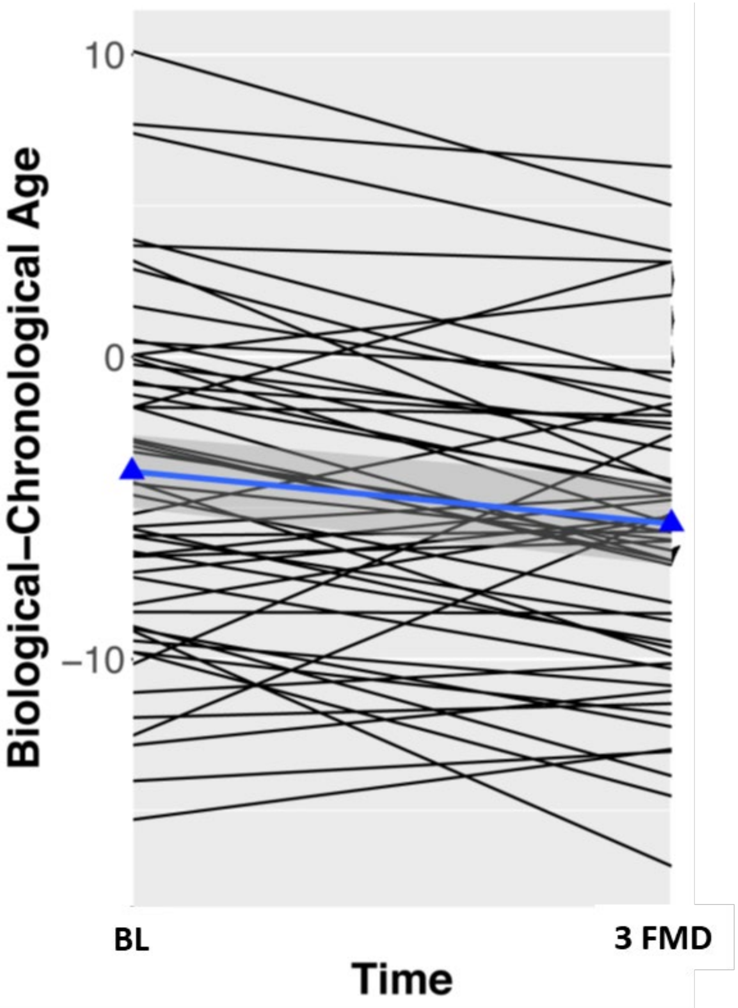

Spaghetti plot for individual (n=51) changes in biological-chronological age between baseline (BL) and after 3 FMD cycles. Blue line indicates the mean change, gray band indicates the SEM.

Supplementary Figure 3: Associations between biological age and individual biomarkers.

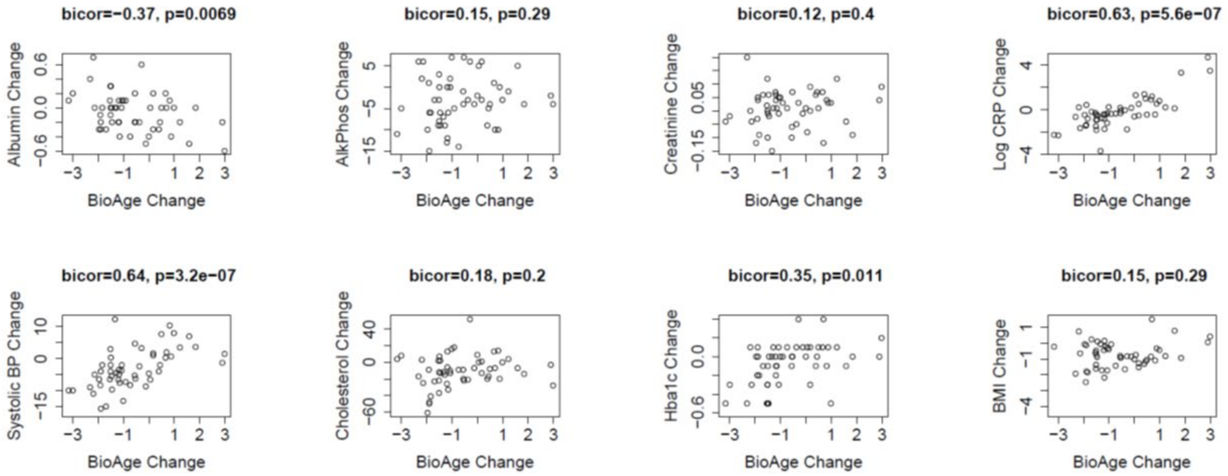

The association between the change in biological age and in the individual biomarkers that make up the biological age estimates to determine if changes in a single marker are driving the changes in biological age. Median-based biweight midcorrelation was used to test for association. Significance was assessed with unadjusted two-sided p-values.

Supplementary Figure 4: Associations between BMI changes and individual biomarkers in at-risk study participants.

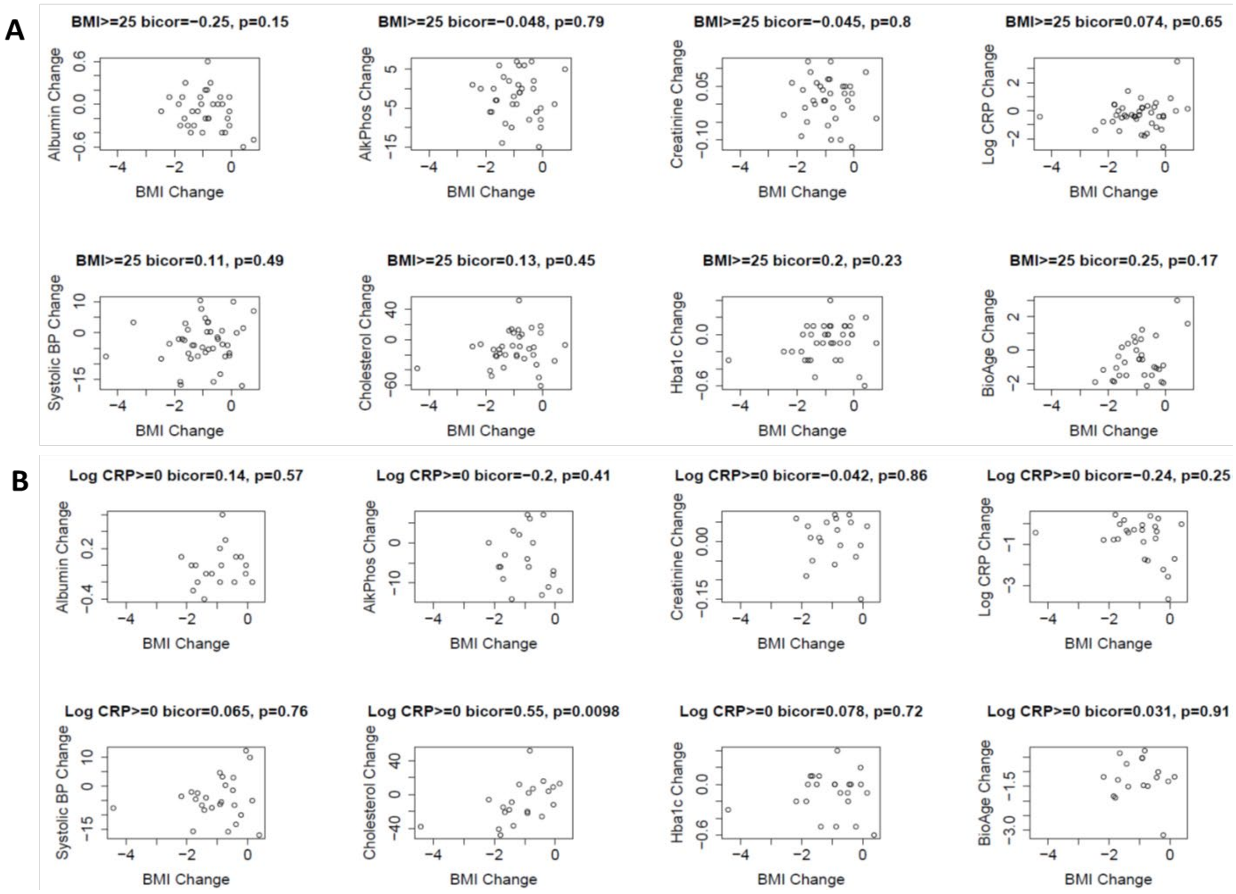

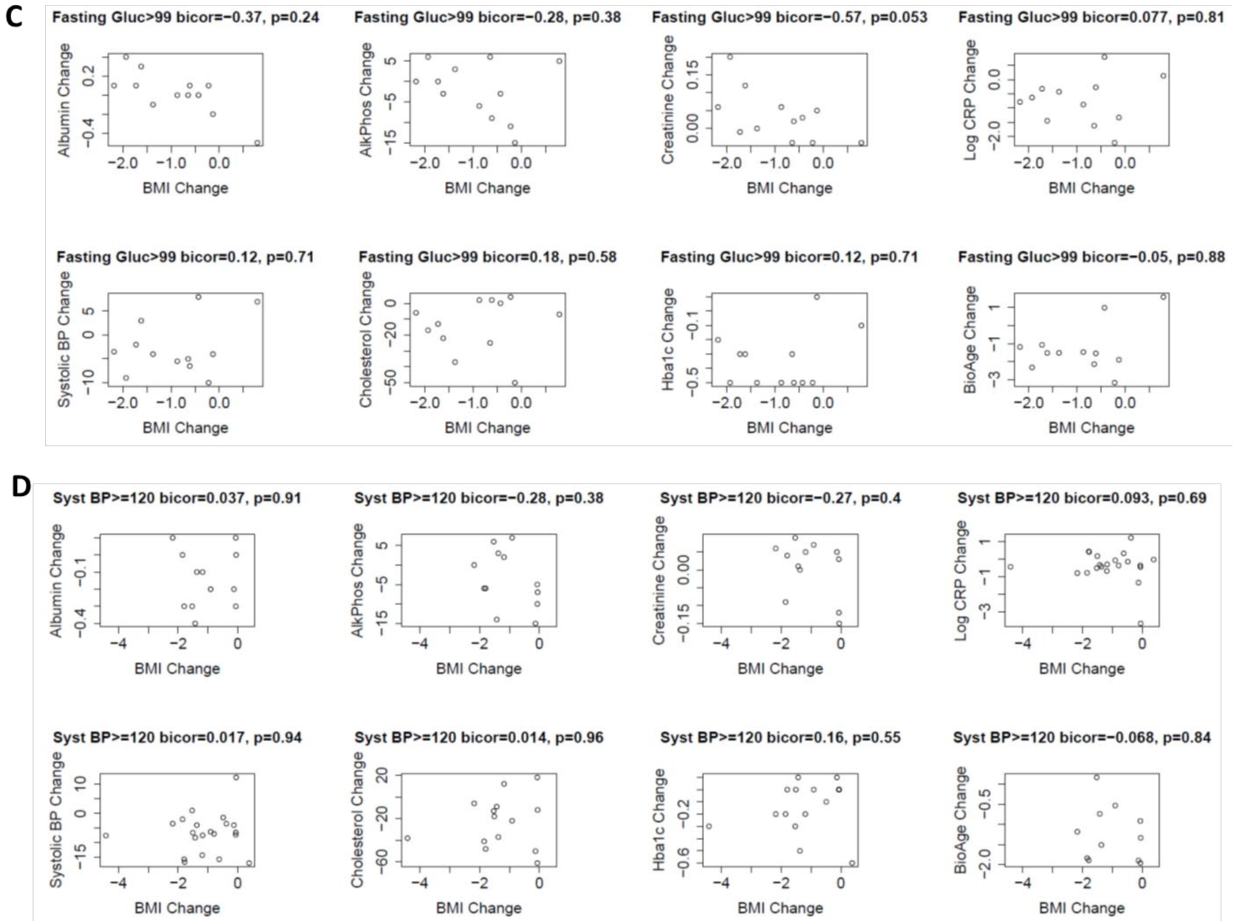

The association between BMI changes and the individual biomarkers in the at-risk study participants defined as having at least one of the following: **A**) BMI >25, **B**) elevated CRP, **C**) fasting glucose >99, and **D**) systolic hypertension. Median-based biweight midcorrelation was used to test for association. Significance was assessed with unadjusted two-sided p-values.

Supplementary Figure 5: FMD increases predicted life expectancy and decreases cause-specific mortality.

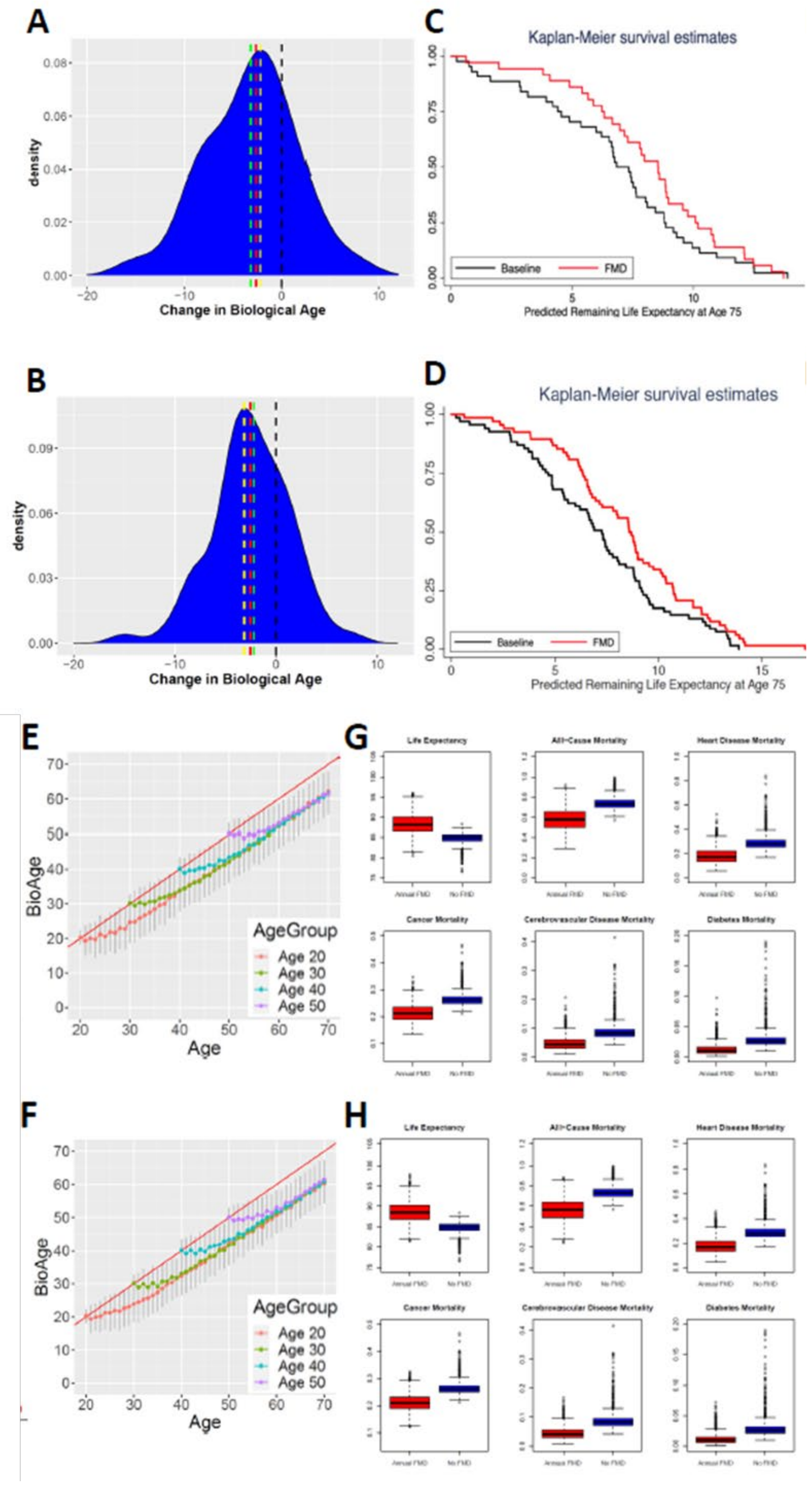

A) In study NCT04150159 and **B**) in pooled samples from both the NCT02158897 and NCT04150159 trials, the average biological age decrease (green line), median decrease (red line), and modal decrease (yellow line) are shown. Predicted median life expectancy based on chronological and biological ages was increased following three cycles of FMD in study **C**) NCT04150159 and **D**) pooled samples from both the NCT02158897 and NCT04150159 trials. The Kaplan-Meier plot depicts predicted survival assuming an age of death equivalent to predicted median life expectancy based on each study participants biological and chronological age at Baseline and following 3 cycles of FMD. Biological Age projection for **E**) study NCT04150159 (N=34 biologically independent samples) and **F**) in pooled samples from both the NCT02158897 and NCT04150159 trials (N=85 biologically independent samples) assuming that people ages 20, 30, 40, and 50 would undergo 3 FMD cycles/year until they reach age 70. The red line indicates if every year the person gained 1 year of biological age and 1 year of chronological age. Mean ± SEM. Median life expectancies and 20-year cause-specific mortality risks based on models from NHANES III assuming that participants had performed 3 cycles of FMD annually (red), or that participants did not undergo FMD (blue) in **G**) study NCT04150159 and in **H**) pooled samples from both the NCT02158897 and NCT04150159 trials. Center line = median, box bounds= interquartile range (IQR, 25th and 75th percentile), whiskers are 1.5xIQR.

Supplementary Figure 6: FMD reduces biological age.

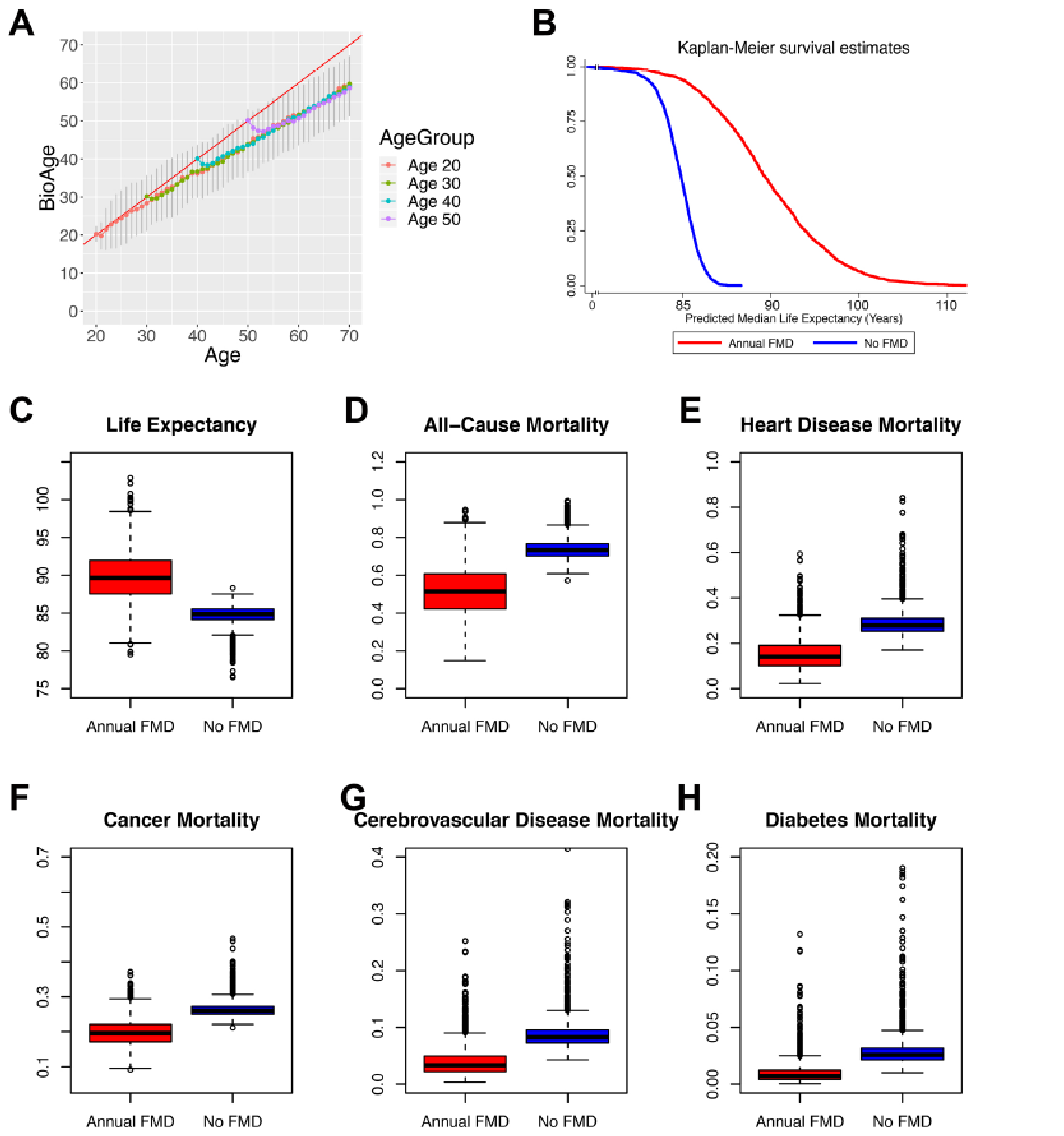

A) Biological Age projection assuming that people ages 20, 30, 40, and 50 would undergo 3 FMD cycles/year until they reach age 70. The red line indicates if every year the person gained 1 year of biological age and 1 year of chronological age chronological age. Mean ± SEM. **B**) Kaplan-Meier life expectancy estimated at age 70, assuming either no FMD (blue) vs. FMD starting at age 50 (red). After aging participants (based on N=51 biologically independent samples) in these simulations up to age 70 (chronologically), we estimated their **C**) median life expectancies, and **D**)-**H**) 20-year cause-specific mortality risks based on models from NHANES III assuming that participants had performed 3 cycles of FMD annually (red) or that participants did not undergo FMD (blue). Center line = median, box bounds= interquartile range (IQR, 25th and 75th percentile), whiskers are 1.5xIQR.
