# Supplementary material for: Fasting-mimicking diet causes hepatic and blood markers changes indicating reduced biological age and disease risk
Source: Nat Commun. 2024 Feb 20;15:1309. doi: 10.1038/s41467-024-45260-9 (PMC10879164; doi:10.1038/s41467-024-45260-9)
Supplement: Supplementary file 2 — Reporting Summary [file 41467_2024_45260_MOESM2_ESM.pdf]

nature portfolio

Corresponding author(s): Valter D. Longo

Last updated by author(s): 12-28-2023

Reporting Summary

Nature Portfolio wishes to improve the reproducibility of the work that we publish. This form provides structure for consistency and transparency in reporting. For further information on Nature Portfolio policies, see our Editorial Policies and the Editorial Policy Checklist.

Statistics

For all statistical analyses, confirm that the following items are present in the figure legend, table legend, main text, or Methods section.

n/a	Confirmed	
☐	☒	The exact sample size (*n*) for each experimental group/condition, given as a discrete number and unit of measurement
☐	☒	A statement on whether measurements were taken from distinct samples or whether the same sample was measured repeatedly
☐	☒	The statistical test(s) used AND whether they are one- or two-sided<br>*Only common tests should be described solely by name; describe more complex techniques in the Methods section.*
☐	☒	A description of all covariates tested
☐	☒	A description of any assumptions or corrections, such as tests of normality and adjustment for multiple comparisons
☐	☒	A full description of the statistical parameters including central tendency (e.g. means) or other basic estimates (e.g. regression coefficient) AND variation (e.g. standard deviation) or associated estimates of uncertainty (e.g. confidence intervals)
☐	☒	For null hypothesis testing, the test statistic (e.g. *F*, *t*, *r*) with confidence intervals, effect sizes, degrees of freedom and *P* value noted<br>*Give P values as exact values whenever suitable.*
☒	☐	For Bayesian analysis, information on the choice of priors and Markov chain Monte Carlo settings
☒	☐	For hierarchical and complex designs, identification of the appropriate level for tests and full reporting of outcomes
☒	☐	Estimates of effect sizes (e.g. Cohen's *d*, Pearson's *r*), indicating how they were calculated

Our web collection on statistics for biologists contains articles on many of the points above.

Software and code

Policy information about availability of computer code

Data collection	Custom code by Morgan Levine. The codes used in the current study are included in the manuscript and also available from the corresponding author on reasonable request.
Data analysis	No software was used.

For manuscripts utilizing custom algorithms or software that are central to the research but not yet described in published literature, software must be made available to editors and reviewers. We strongly encourage code deposition in a community repository (e.g. GitHub). See the Nature Portfolio guidelines for submitting code & software for further information.

Data

Policy information about availability of data

All manuscripts must include a data availability statement. This statement should provide the following information, where applicable:

- Accession codes, unique identifiers, or web links for publicly available datasets
- A description of any restrictions on data availability
- For clinical datasets or third party data, please ensure that the statement adheres to our policy

The datasets generated during and/or analyzed during the current study are deposited using FigShare uplaods and are also available from the corresponding author on reasonable request.

Field-specific reporting

Please select the one below that is the best fit for your research. If you are not sure, read the appropriate sections before making your selection.

- [x] Life sciences
- [ ] Behavioural & social sciences
- [ ] Ecological, evolutionary & environmental sciences

For a reference copy of the document with all sections, see nature.com/documents/nr-reporting-summary-flat.pdf

Life sciences study design

All studies must disclose on these points even when the disclosure is negative.

Sample size	As previously reported, for this randomized trial, the sample size of 100 total subjects was based on the detection of a 25% reduction in mean insulin-like growth factor-1 (IGF-1), with a two-sided α of 0.05 and 70% power. The estimated control group mean (SD) IGF-1 of 194 (97) used published data on males and females aged 26 to 40 years
Data exclusions	Only subjects that completed all FMD cycles were used. Dropouts were excluded.
Replication	Similar results were replicated using data from a second clinical trial (NCT04150159)
Randomization	Eligible participants were randomly assigned using a random-number generator to either arm of the study
Blinding	All authors, except MV and SB, had access only to blinded and deidentified data.

Reporting for specific materials, systems and methods

We require information from authors about some types of materials, experimental systems and methods used in many studies. Here, indicate whether each material, system or method listed is relevant to your study. If you are not sure if a list item applies to your research, read the appropriate section before selecting a response.

Materials & experimental systems

n/a	Involved in the study	
x		Antibodies
x		Eukaryotic cell lines
x		Palaeontology and archaeology
x		Animals and other organisms
	x	Human research participants
	x	Clinical data
x		Dual use research of concern

Methods

n/a	Involved in the study	
x		ChIP-seq
x		Flow cytometry
x		MRI-based neuroimaging

Human research participants

Policy information about studies involving human research participants

Population characteristics	The baseline characteristics at enrollment are presented in Table 1 of the manuscript as well as in Wei et al. Science Translational Medicine, 2017. Both arms were comparable for age, sex, race, and body weight.
Recruitment	All data were collected at the USC Diabetes and Obesity Research Institute. Subjects were recruited throughout Los Angeles County and surrounding areas from April 2013 to July 2015 based on established inclusion (generally healthy adult volunteers and 18 to 70 years of age; BMI, 18.5 and up) and exclusion [any major medical condition or chronic diseases, mental illness, drug dependency, hormone replacement therapy (dehydroepiandrosterone, estrogen, thyroid, and testosterone), pregnant or nursing female, special dietary requirements or food allergies, alcohol dependency, and medications known to affect body weight] criteria.
Ethics oversight	The University of Southern California (USC) Institutional Review Board ; HS-12-00391

Note that full information on the approval of the study protocol must also be provided in the manuscript.

Clinical data

Policy information about clinical studies

All manuscripts should comply with the ICMJEguidelines for publication of clinical research and a completedCONSORT checklist must be included with all submissions.

Clinical trial registration	NCT02158897; NCT04150159

Study protocol	The complete study protocol is available in Wei et al. Science Translational Medicine, 2017
Data collection	Subjects were recruited from April 2013 until July 2015
Outcomes	Pre-specified outcome measures include safety and feasibility, and evaluation of changes in risk factors for metabolic syndrome and biomarkers associated with age-related diseases and mortality during and after completion of the intervention. Primary outcome measures were reported before. Here we performed a secondary as well as exploratory analysis of available data.
